# Evidence of protein-free homology recognition in magnetic bead force–extension experiments

**DOI:** 10.1098/rspa.2016.0186

**Published:** 2016-07

**Authors:** D. J. (O’) Lee, C. Danilowicz, C. Rochester, A. A. Kornyshev, M. Prentiss

**Affiliations:** 1 Department of Chemistry, Imperial College London, London SW7 2AZ, UK; 2 Department of Physics, Harvard University, Cambridge, MA 02138, USA

**Keywords:** DNA, homology recognition, single-molecule force experiments, molecular interactions, macromolecular mechanics

## Abstract

Earlier theoretical studies have proposed that the homology-dependent pairing of large tracts of dsDNA may be due to physical interactions between homologous regions. Such interactions could contribute to the sequence-dependent pairing of chromosome regions that may occur in the presence or the absence of double-strand breaks. Several experiments have indicated the recognition of homologous sequences in pure electrolytic solutions without proteins. Here, we report single-molecule force experiments with a designed 60 kb long dsDNA construct; one end attached to a solid surface and the other end to a magnetic bead. The 60 kb constructs contain two 10 kb long homologous tracts oriented head to head, so that their sequences match if the two tracts fold on each other. The distance between the bead and the surface is measured as a function of the force applied to the bead. At low forces, the construct molecules extend substantially less than normal, control dsDNA, indicating the existence of preferential interaction between the homologous regions. The force increase causes no abrupt but continuous unfolding of the paired homologous regions. Simple semi-phenomenological models of the unfolding mechanics are proposed, and their predictions are compared with the data.

## 1. Introduction

The pairing of homologous chromosomes in the absence of DNA breakage is an essential step in many biological processes [1]. It has been established that proteins such as cohesin, condensin, CCCTC-binding factor (CTCF) and others have a role in promoting, or maintaining, sequence-dependent pairing [1,2]. Here, we focus on the possibility that direct physical interactions between DNA molecules might also be important. Such interactions may provide a mechanism for pairing in the absence of recognition proteins, and they may facilitate pairing in the presence of proteins. Indeed, experimental studies of protein-independent homologous pairing between dsDNA have begun to attract increasing interest in the biology community [3,4]. Notably, Barzel & Kupiec [4] asked the question whether the pairing of homologous sequences could be an innate general characteristic of the genome. Similar queries were also raised and discussed by Zickler [3].

The previous experimental work has studied homology-dependent dsDNA interactions in a protein-free environment [5–8]. Homology-dependent pairing has been directly observed at a single-molecule level [7], as well as phase segregation in aggregates [6] between two types of DNA molecules with different base-pair sequences.

It is quite possible that both homology-dependent and -independent interactions play roles *in vivo* and are interlinked to facilitate homologous pairing. Homology-independent dsDNA–dsDNA forces contribute to the formation of viral capsids [9,10]. Attractive interactions between plectonemic supercoils may play an important role in creating and stabilizing chromosome superstructures that have been observed in bacteria [11], whereas interactions within supercoils determine their average structure, as highlighted in theoretical and single-molecule experimental studies [12–16].

The valence and species of ions surrounding the DNA, large negative charge on the phosphate backbones, and the loss of dsDNA conformational entropy are all important in determining dsDNA–dsDNA interactions. In solutions containing multivalent ions, there can be strong attractive homology insensitive electrostatic interactions between dsDNA molecules [17,18]; explanations include correlation effects between fluctuations in the local counterion charge densities [19] and ordering of multivalent ions near the surfaces of DNA duplexes [9,20]. Both possibilities create a negative–positive charge motif that depends on the counterion valence and salt concentration. However, ion-specific effects are needed to explain why cobalt-hexamine is far more efficient in condensing DNA than spermine [17], and why divalent ions generally do not condense dsDNA, but manganese ions sometimes do [21].

In the absence of multivalent salts, there is less understanding of how attractive interactions between dsDNA could emerge. Two possible entropic origins that do not necessarily depend on homology are: (i) molecular crowding, which will push dsDNA molecules together and align them parallel to each other, and (ii) the release of bound water, ions or ion–water clusters that are freed as a result of the pairing of the dsDNA. In addition, recent simulations [22] reveal a higher than expected degree of localization of monovalent counterions with their distribution between the major and minor groove being sequence specific.

The role of the helical structure of the dsDNA molecules is also important for our discussion. Theory predicts the existence of correlations in the relative azimuthal orientations of neighbouring molecules about their long axes [23]. There is an optimal alignment where the pattern of negative charges of phosphates and positive charges of adsorbed/condensed counterions, tracing out the DNA helicity, aligns preferentially with that of another molecule. This has been shown for mean-field electrostatics [20] and for correlation forces modulated by the helically distributed fixed charges [24,25]. Evidence of azimuthal correlations between DNA molecules are seen in the X-ray diffraction patterns of DNA assemblies [17,26], and in cryo-electron micrograph cross-sections of DNA toroids [10].

There are two ways that homology-dependent pairing interactions between two dsDNA molecules might depend on their helical structure. One mechanism relies on similarities (dissimilarities) of the base-pair sequence-dependent distortions of the DNA double helices [27–29], making a pair of homologous molecules have a lower interaction-free energy than that between two non-homologous ones [30–33]. A second possibility is a pairing interaction that depends locally on base-pair sequence. This should also depend on the relative orientations of the two base pairs in question, which in turn relies upon helical structure. A candidate for this is the sequence-dependent localization of ions [22] between the facing grooves of the two molecules. Molecular dynamic studies [34] suggest that this might play a role, at least for two poly (AT) dsDNA molecules in the presence of monovalent salts. Global and local mechanisms are not mutually exclusive, and both may be instrumental in homology-dependent pairing.

The previous single-molecule work studied the probability that two separate dsDNA molecules would come together to form a single structure [7]. In those experiments, the pairing was observed and measured in real time. Now, we perform experiments, in which we observe the pairing *between two regions of a single dsDNA molecule* that contains the same sequence linked head to head. Using magnetic tweezers that apply force to the dsDNA, we are able to allow the regions to pair and then pull them apart. Thus, we can repeatedly monitor the pairing of two regions within the same dsDNA molecule. In what follows, we rationalize the results of these experiments by simple coarse-grain models and relate them with different mechanisms for dsDNA pairing. Importantly, the results suggest that the pairing energy is not a linear function of the paired length.

## 2. Material and methods

### (a) Preparation of DNA construct

Briefly, the fragments were amplified using Pfu Ultra II fusion (Stratagene, Carlsbad, CA, USA) in a thermocycler. Typical conditions were as follows: 1 ng λ-phage dsDNA, PFu Ultra II buffer, 1 mM MgCl_2_, 0.5 μM dNTPs and 1 Unit Pfu Ultra II fusion were mixed in a total of 50 μl. The cycling protocol was 5 min at 95°C, 30 cycles of 30 s at 95°C, 30 s at 55°C, 3 min at 72°C and 15 min at 72°C using primers to amplify between positions 16 322 and 26 598 on λ DNA. One of the primers was 5′ labelled with digoxigenin, while the other primer contained OMe bases that separated a 12-mer region complementary to one of the cos-segments in λ phage. Following PCR, the fragments were separated via gel electrophoresis on a 1× TRIS/borate/EDTA (TBE) buffer 1% agarose gel. The fragments were gel purified using a Nucleospin kit (Machery and Nagel, Bethlehem, PA, USA), subsequently annealed and ligated to λ-phage DNA biotinylated at one end. λ-phage dsDNA was previously hybridized and ligated to an oligonucleotide complementary to the ssDNA tail at the left end of λ that contained a biotin-label. An aliquot of the sample containing the construct was incubated for 2 min with superparamagnetic (Dynal 2.8 μm diameter) antidigoxigenin-coated beads, placed for 10 min in a micro-channel with square cross-section 0.8 mm, containing a round capillary, 0.55 mm diameter, previously coated with Extravidin. During this latter step, digoxigenin-labelled ends of the molecules became tethered to the surface of the magnetic beads while the biotinylated ends of the molecules remained associated with the capillary. In most of our experiments, we used phosphate-buffered saline (PBS) containing 150 mM NaCl. We also did some experiments at NaCl concentrations ranging from 100 mM to 3 M. Finally, we did a few experiments in buffers containing 0.4–2 mM MgCl_2_.

### (b) Apparatus

The magnetic field gradient is produced by one stack of five permanent magnets each of 6.4×6.4×2.5 mm^3^ dimensions. The magnets were held in a lateral position with respect to the micro-channel containing the sample and exerted a force perpendicular to the glass surface to which the DNA was bound at one end and the magnetic beads attached to the DNA at the other end. The micro-channels were placed on a sample holder whose position was controlled by a 3-axis translation. The magnitude of the force applied on the beads was controlled by the distance between the magnet and the glass surface. The temperature was controlled by a thermoelectric cooler that allowed us to do experiments at temperatures from 20°C to 45°C.

## 3. Results

### (a) Experimental approach

**Figure 1.**
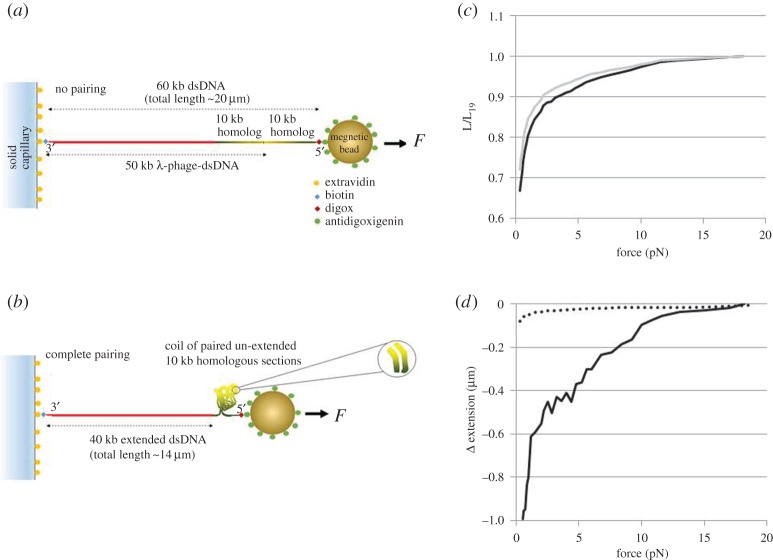
Schematic of the looped dsDNA unfolding experiments for the case of (*a*) no pairing and (*b*) complete pairing. The construct is built from 50 kb λ-phage dsDNA ligated at the end to a 10 kb homologous fragment (for details see the main text). Here, the red lines represent the part of dsDNA which is not identical in sequence to the 10 kb fragment. The two 10 kb sequences which are identical to each other are shown in green to yellow shading to indicate which way the two sequences run (the yellow indicating the end sequence of the λ-phage DNA). When the constructs are fully extended the two homologous pieces run in opposite directions, but when folded any paired tracts will run in the same direction. Shown in (*b*) is a coil–expected form for the DNA – where the magnification shows that, where pairing occurs, two sections may align so that their identical sequences read in the same direction. (*c*) Is a representative result showing the extension versus force curves (decreasing force only) in 150 mM NaCl–PBS at 25°C for a dsDNA construct (solid) and a control curve for a 60 kb dsDNA in the same ionic conditions (dashed). The *y*-axis is normalized to L_19_, the measured extension of the controls at 19 pN. Further experimental data curves for different ionic conditions are given in the electronic supplementary material, S1. (*d*) The solid line shows the difference between the two curves (here, defined as the extension of the construct minus the experimentally measured control) shown in (*c*), multiplied by *L*_19_, resulting in negative values since the extension of the paired dsDNA is shorter than the 60 kb control. The dotted line shows the standard deviation for 25 controls. At forces less than 2 pN, the constructs containing homologous regions show significant variation, but even at 5 pN the difference between the constructs and controls is more than 10 times the standard deviation in the controls. The results indicate a significant interaction between identical DNA tracts, which affects the extension–force curve even when the applied force exceeds 10 pN. Experiments for higher temperatures (37°C and 40°C) have also been performed that are not shown here; they demonstrate qualitatively similar effect of ‘homology recognition’; the data for these temperatures are treated in the electronic supplementary material, S5. (Online version in colour.)

The dsDNA constructs used to study the extent of the pairing were prepared by ligating λ-phage dsDNA to a sequence matched 10 kb fragment from the end of the λ-phage DNA (figure 1*a*,*b*). If the dsDNA completely pairs over the entire homologous region, then the two 10 kb regions will fold together as shown in figure 1*b*. In order to detect this pairing, we specifically attached one end of the dsDNA to a capillary surface and the other end, corresponding to the ligated fragment, to a magnetic bead.

Experimentally, we measure the extension as the distance between the centre surface of the bead and the edge of the capillary surface when both are in focus. We obtain the dsDNA length by subtracting the radius of the bead from the measured distance. When we fit theoretical curves to the data, we include a constant offset in the curves. If the dsDNA does not pair at all, the distance between the bead and the surface with respect to the pulling force can be described by the worm-like chain (WLC) model [35–41], corresponding to the chosen molecular length of 60 kb. By contrast, if the homologous region of the dsDNA completely pairs, only 40 kb of dsDNA length would contribute to the extension (figure 1*b*). Partial pairing results in an extension somewhere between these values. Thus, for a given applied force, we can quantify the extent of the pairing by measuring the distance between the magnetic bead and the surface of the capillary, and then subtracting the extension for 60 kb dsDNA containing no identical sequences (the control experiment). If there is no pairing, the difference will be zero. By comparing the extension differences for various applied forces, we can determine the force dependence of pairing.

If the homologous dsDNA was completely zipped along its entire length, then the paired region would be equal to the length of the loop—the measured difference between construct and control dsDNA of the same length. If the interactions between the homologous tracts are localized, then the paired length may be smaller than that difference, and the pairings may change with time without producing any significant change in the measured extension.

Similar to the well-known force-induced intra DNA unzipping ‘bubbles’, the presence of bubbles in the dsDNA loop influences the free energy of the system. The distribution of the bubbles may change significantly with time, but this does not affect fluctuations in the force-dependent extension, if the bubbles are small and sparse.

For the dsDNA–dsDNA interactions discussed here, the paired regions themselves may interact forming additional higher order structures. In that case, the interpretation of the measured extension is even more complicated; however, the important point is that if the extension is less than the one for the control experiments then some pairing must occur. Furthermore, if that extension difference depends on the presence of homologous sequences within the construct molecules, then favourable homology-dependent interactions are only responsible for this. This fact itself is not reliant on any particular model for the homology pairing.

### (b) Initial findings

We initially performed control experiments where the 10 kb fragment is not homologous to any part of the 50 kb of the λ-phage dsDNA. The force distance curves obtained for the 60 kb dsDNA control experiments fit the WLC extension law (of ref. [41], as was verified in experiments [35]), indicating that no non-homologous attractive intersegment interactions were present. By contrast, when we performed the same experiments in the same ionic environment with the inserted homologous 10 kb fragment, we found that at forces less than 10 pN the measured extension was significantly less than the extension for the control. For higher applied forces, the homologous segments are unpaired, as no significant extension difference was observed. One representative difference in the measured extension versus force curves between homologous and non-homologous constructs is shown in figure 1*c*,*d*; for more examples, see the electronic supplementary material, figure S1. These results demonstrate also that at low forces (less than 10 pN) the extent of pairing is a smooth continuous function of the applied force with little fluctuation in extension difference. Under some conditions, at high forces (more than 15 pN) increasing force curves showed a brief slope increase. Force measurements made in buffers containing MgCl_2_ concentrations of 0.4–2 mM are qualitatively similar to results made in 150 mM NaCl, though the high-force regime for MgCl_2_ began at approximately 5 pN, whereas the high-force regime in NaCl began at more than 10 pN.

The observed difference between the constructs and controls does not seem to be accounted for by sequence-dependent effects in the DNA elasticity. According to numerous experimental [35,36] and theoretical studies [37,38], the bending elasticity over large length scales, for genomic DNA, is in fact sequence independent (even for relatively short molecules, the elastic response of the DNA molecules was found to behave in WLC fashion [39,40]). The tracts of DNA considered here are indeed sufficiently long to neglect sequence-dependent effects. Furthermore, we did not introduce any new sequences, rather we PCR-amplified one piece of the λ-phage dsDNA used for the control experiments. In addition, the difference is not an artefact due to experimental uncertainty of the bending persistence length. In figure 1, and figure S1 of the electronic supplementary material, we show that differences in the control curves are indeed much smaller than the difference between the construct and the control. All of this indicates the preferential pairing between the identical sequences.

### (c) General theoretical considerations

To model features seen in the experimental data, we first divide the length of the dsDNA into regions of two conformational types. In the first one, the dsDNA is extended and does not interact with itself (figure 1*a*). In the regions of the second type the dsDNA is looped, with the identical, parallel-oriented tracts interacting with each other. The extended regions can be described by the WLC model, for which the extension–force dependence is well known; we assume that only these regions contribute to the extension. As the force is controlled by the magnets, we deal with a fixed force ensemble and consider fluctuations in the extension. For a DNA molecule of length *L*, on which a pulling force *f* is acting, we may write the total free energy, *F*_*T*_, as

3.1 $$F_{T}=g_{\mathrm{wlc}}(\;f)(L-2L_{\mathrm{loop}})+F_{\mathrm{loop}}(L_{\mathrm{loop}}),$$

where 2*L*_loop_ is the contour length of the looped region. Here, *g*_wlc_(*f*) is the WLC free-energy density. For sufficiently large pulling forces *f*(>1 pN), it is described by [41]:

3.2 $$g_{\mathrm{wlc}}(\;f)=-f+\sqrt{\frac{k_{B}Tf}{l_{p}}},$$

where *l*_p_ is the bending persistence length. The second term in this expression is the correction given by WLC fluctuations; strictly speaking, it must be small for equation (3.2) to be valid. Relative to the first term, the contribution of this correction grows as the pulling force decreases, and at forces below 1 pN equation (3.2) can no-longer be considered valid. However, for the experiments of this paper, the applied values of force are sufficiently large for this equation to apply. For estimating the parameter dependence of the models presented below, we fix *l*_p_ to its typical value of 500 Å, but when analysing the experimental data, we fit *l*_p_ to the force–extension curves of the control experiments for different salt concentrations. Indeed, the value of *l*_p_ is known to change with salt concentration [35], as well as other factors in the pulling experiments. We adopt those values, obtained for each salt concentration when treating the data for the difference in force–extension curves between constructs and controls.

From equations (3.1) and (3.2) for the constant force ensemble, it follows that at sufficiently large pulling force the average extension of the molecule is given by:

3.3 $$z=-\frac{dF_{T}}{df}=\left(1-\sqrt{\frac{k_{B}T}{4l_{p}f}}\right)(L-2L_{\mathrm{loop}}).$$

For DNA in the control experiments, *L*_loop_=0 and we recover the classic WLC extension formula [41]. Thus, in this model, the difference in extension between the constructs and the controls lies in a non-zero value of *L*_loop_. This value may be found from minimizing the total free energy (equation (3.1)).

We first demonstrate that the simplest and most ‘obvious’ choice for the form of *F*_loop_(*L*_loop_) will fail to describe the experimental observations reported in this paper. Indeed, let us consider *F*_loop_(*L*_loop_)=*ε*_loop_*L*_loop_, where *ε*_loop_ is the pairing free energy per unit length for the DNA in the loop. In this case, for a loop to form, we need *ε*_loop_<0. As in Lubensky & Nelson [42] and other studies, in the first approximation, *ε*_loop_ can be assumed force independent, provided that the paired state is a sharp minimum in the energy as a function of the separation between segments. Corrections to this assumption could be included by writing *ε*_loop_ as a Taylor series in *f*, if the response of *ε*_loop_ is known, but such corrections are likely to be small, and the loop may become unstable before these corrections become significant. The linear dependence of the free energy on *L*_loop_ would lead to an abrupt transition at a critical pulling force *f*_c_. Indeed, when −2*g*_wlc_(*f*)>−*ε*_loop_ the linear form of free energy in *L*_loop_ suggests that *L*_loop_ should vanish to minimize it. When −2*g*_wlc_(*f*)<−*ε*_loop_, *L*_loop_ should acquire its maximum possible value, which is the full length of the homologous segments, *L*_max_. Thus the jump-wise change in the extension from fully looped to fully extended would take place at a pulling force larger than a critical value, *f*_c_, which is defined by equation, *ε*_loop_=2*g*_wlc_(*f*_c_). But this is not what we see experimentally. A more careful consideration is therefore needed to build a working model.

One could try including thermal fluctuations in *L*_loop_. As *f* approaches *f*_c_, the fluctuations in the value of *L*_loop_ and in the overall measured extension will increase drastically. Fluctuations of *L*_loop_ will make its average value, 〈*L*_loop_〉, vary smoothly with the applied force, as opposed to a sudden jump from $L_{\max}$ to 0. Indeed, if we average *L*_loop_ over the Boltzmann distribution $W(L_{\mathrm{loop}})=\exp\{-[\varepsilon_{\mathrm{loop}}-2g_{\mathrm{wlc}}(f)]L_{\mathrm{loop}}/k_{B}T\},$ we obtain 〈*L*_loop_〉≈*k*_B_*T*/(*ε*_loop_−2*g*_wlc_(*f*)). To have a significantly large, changing value of *L*_loop_ the pulling force *f* must be close to *f*_c_. Then, for *f*>*f*_c_, this expression reduces to 〈*L*_loop_〉≈*k*_B_*T*/(*f*−*f*_c_), a finite value that decreases continuously with *f*.

The latter expression, however, formally diverges at *f*=*f*_c_, whereas it should not be larger than the length of the homologous identical segments. This is because, in deriving the above expression for 〈*L*_loop_〉, we have assumed that the range of integration is from 0 to ∞. Strictly speaking, the limits of integration should be from 0 to $L_{\max}$ resulting in a more complicated expression for 〈*L*_loop_〉. But for relevant values of the force, except within a very narrow range close to *f*_c_, $L_{\max}\left|\varepsilon_{\mathrm{loop}}-2g_{\mathrm{wlc}}(f)\right|/k_{B}T\gg 1$. In our experiments, $L_{\max}\approx 34\;000$ Å. Thus, 〈*L*_loop_〉 can be, in fact, well approximated by $\left\langle L_{\mathrm{loop}}\right\rangle \approx \min\left(k_{B}T/(\;f-f_{c}),L_{\max}\right)$. (The consequences of the assumption of a linear dependence of the free energy on *L*_loop_ have been discussed in the context of DNA unzipping [42].)

To be resolved in the experiments, a typical value of *L*_loop_ should be no less than the order of 1000 Å. Consequently, to account for the observations reported in this paper, *f*−*f*_c_ must be of the order of 10^−3^ *k*_B_*T*/ Å. In the experimental data, significant, diminishing values of *L*_loop_ seem to persist over a much larger range of force values (to appreciate this, note that 10 pN ≈ 0.25 *k*_B_*T*/ Å). In addition, if thermal fluctuations near *f*_c_ were responsible for the continuous transition observed, we would have also expected more noise in the force extension data, as the amplitude of fluctuations is governed by Δ*L*_loop_=(〈*L*^2^_loop_〉−〈*L*_loop_〉^2^)^1/2^, where 〈*L*^2^_loop_〉≈2(*k*_B_*T*)^2^/(*ε*_loop_−2*g*_wlc_(*f*))^2^≈ 2(*k*_B_*T*)^2^/(*f*−*f*_c_)^2^. But, we do not see this in our experimental results. Because there is no critical force, the free energy of the loop, *F*_loop_, must depend nonlinearly on *L*_loop_. With this in mind, we were led to consider two alternative approaches, each of which can rationalize the experimental data.

### (d) Model 1

One way to have a nonlinear dependence of *F*_loop_ on *L*_loop_ is to include some kind of collective interaction between different homologously paired regions of the loop. Such interactions could form transient higher order structures that would readjust as the loop length changes, creating equilibrium lengths that change continuously with the variation in the applied force. The favourable interactions between homologously paired regions would help stabilize the loop, i.e. they provide a reduction of free energy, once homologously paired regions have formed. If the interaction is short ranged, its effective strength should depend on the square of the local density of homologously paired DNA. For simplicity, we assume that the homologously paired length is proportional to *L*_loop_. When the loop is very long, polymer scaling arguments (see the electronic supplementary material, S2) suggest that the strength of the interaction between homologously paired regions scales as *E*_int_∼*L*^2−3*ν*^_loop_. The exponent *ν* is normally taken to be close to 3/5 to account for excluded volume effects [43,44]. However, here, attraction may compensate the repulsion; thus, we assume a random walk exponent of 1/2, resulting in *E*_int_∼*L*^1/2^_loop_ that we will use hereafter.

In addition, one needs to consider the bending energy cost for paired regions of the loop to come close to each other. We can take this into account by introducing another *L*_loop_-dependent factor, exp(−*b*/*L*_loop_). Such a term increases the loop-free energy. When *L*_loop_ becomes too small, the curving back of the loop to interact with itself will cost too much bending energy. The phenomenological parameter *b* is of the order of a bending persistence length. This factor prevents an unphysical divergence of the effective interaction strength per unit length (∝*L*^−1/2^_loop_), as we make the loop smaller.

Altogether, based on these considerations, we write a new modified expression for the looping free energy

3.4 $$F_{\mathrm{loop}}(L_{\mathrm{loop}})=\varepsilon_{\mathrm{loop}}L_{\mathrm{loop}}-aL_{\mathrm{loop}}^{1/2}\exp\left(-\frac{b}{L_{\mathrm{loop}}}\right),$$

which should be incorporated into equation (3.1). In this model, we may even allow for *ε*_loop_>0, which describes a local metastable pairing energy between the two identical segments forming the loop, in the absence of the stabilizing non-local interactions given by the second term. The magnitude of the latter interactions is controlled by the positive parameter *a*. In the electronic supplementary material, Section S4, we plot *F*_loop_(*L*_loop_)−2*g*_wlc_(*F*)*L*_loop_, corresponding to equations (3.1) and (3.4) combined, for a typical set of parameters that illustrate the possibility of stable and metastable looped states.

It is interesting to note that for *ε*_loop_>0, in the absence of a pulling force, equation (3.4) suggests that there is a maximum size to *L*_loop_. Indeed, the maximum value that the loop can take is roughly the length of identical sequences, $L_{\max}$. But if the latter is very long, the density of the favourable collisions may become too small: thus, there will be a maximum possible value of *L*_loop_, independent of the length of the identical sequences. Indeed, if we have *L*_loop_≫*b*, then at *f*=0, *L*_loop_≈(*a*/2*ε*_loop_)^2^, provided that $L_{\mathrm{loop}}<L_{\max}$. As we increase the pulling force, *L*_loop_ decreases significantly until we reach a point where we get a sudden unfolding of the loop, down to *L*_loop_=0. However, the magnitude of both the jump, and this force, depends on the size of the parameter *b*. As we decrease *b*, the jump moves to larger values of the pulling force and it gets smaller (cf. electronic supplementary material, S4). In experimental data, this jump is not seen over the range of forces considered, or it is too small to be resolved. Thus, we choose *b* to take its lowest plausible value which is the Kuhn length [43,44], twice the bending persistence length, to fit the data.

In the case when *ε*_loop_<0, *L*_loop_ is at its maximal value $L_{\max}$ until *f*_c_ is reached. Then, the loop starts to gradually diminish in size as we increase the force; however, at higher forces, the loop again collapses to *L*_loop_=0.

An exact expression for the optimal value of *L*_loop_ cannot be obtained analytically. However, the numerical solution is well approximated by the function given in the electronic supplementary material, S3.

In the interpretation of the experiments, done at a certain rate of increase in the pulling force, we can even allow the *L*_loop_≠0 minimum in the free energy to be metastable. This would imply that the energy barrier for collapse to *L*_loop_=0 is sufficiently large for thermal fluctuations not to cause the disappearance of the loop.

**Figure 2.**
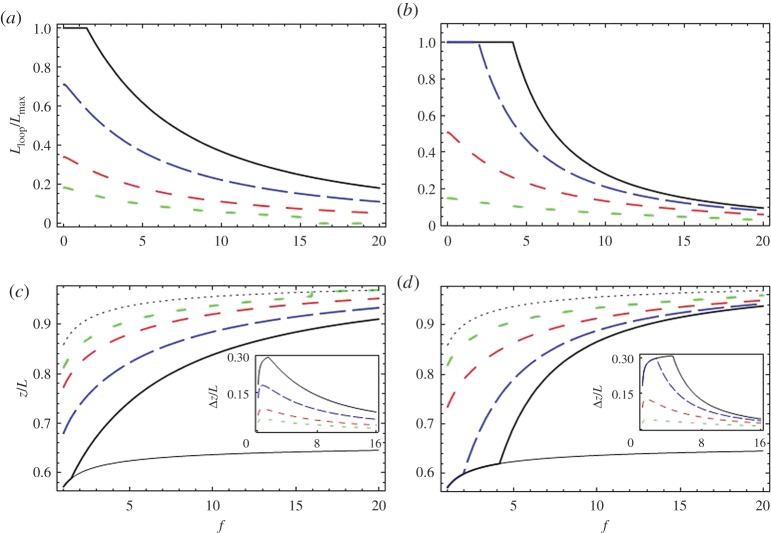
Exploring the effects predicted by model 1. Plots for the length of the looped section, *L*_loop_ and the extension length, *z* (defined in figure 1), as a function of applied force, for various values of the model parameters, calculated using equations (3.1), (3.2) and (3.4). Here, the typical value of *b*=2*l*_p_= 1000 Åis used. Plots of *L*_loop_, normalized over $L_{\max}\approx 34\;000$ Å(the maximum length for pairing of the homologous segments) are shown in (*a*,*b*). Plots of *z* divided by the total DNA length *L* are shown in (*c*,*d*). Here, the thin solid line is the extension–force curve, which would have emerged for total homologous pairing ($L_{\mathrm{loop}}=L_{\max}$); the thin dotted line is that for no pairing (*L*_loop_=0). The insets in (*c*,*d*) show Δ*z*, the difference in extension between the case with no pairing (thin dotted line) and those with homologous pairing. In (*a*,*c*), *ε*_loop_=0.5 *k*_B_*T*/ Å, and the thick solid, long dashed, medium dashed and short dashed lines correspond to the values *a*=200, 150, 100, 70 *k*_B_*T*/ Å^1/2^. In (*b*,*d*), *a*=100 *k*_B_*T*/ Å^1/2^, and the solid, long dashed, medium dashed and short dashed lines correspond to the values *ε*_loop_=0.1,0.2,0.4,0.8 *k*_B_*T*/ Å. (Online version in colour.)

In figure 2, we show both *L*_loop_ and the resulting extensions, *z*, for various values of the model parameters. Both decreasing *ε*_loop_ and increasing *a* increase *L*_loop_ and reduce the extension. In addition, increasing *a* (as well as reducing *b*, which in figure 2 is, however, kept fixed) increases the range of force values over which *L*_loop_ diminishes gradually, before suddenly dropping down to zero. Decreasing *ε*_loop_ shifts this range to higher force values. In all of the plots, the maximum length of the loop is the length of one homologous segment, $L_{\max}=34\;000\;\text{Å}$. In the inset, we show Δ*z*, the difference between the force–extension curves of for non-zero *L*_loop_ and the control (*L*_loop_=0). With increasing *f*, Δ*z* first increases rapidly before gradually decreasing. These features are seen in the experimental data and can be explained by *L*_loop_ monotonically decreasing with increasing force, as well as the WLC behaviour of the unpaired parts of the molecule.

### (e) Model 2

An alternative non-linear form for *F*_loop_ is

3.5 $$F_{\mathrm{loop}}(L_{\mathrm{loop}})=F_{\mathrm{loop}}(L_{0})+\frac{1}{\beta }{\left(L_{0}-L_{\mathrm{loop}}\right)}^{2}.$$

Here, *L*_0_ is the length of *L*_loop_ at zero pulling force and is thus the maximum value of the loop. The second term in equation (3.5) describes an increase in the energy per unit length needed to un-pair the loop, as *L*_loop_ diminishes from its zero force value. Equation (3.5) is the simplest choice of a free energy that has the minimum value *L*_loop_=*L*_0_ at *f*=0. It could be considered as a harmonic approximation to some more complicated form of *F*_loop_(*L*_loop_).

We consider what factors may determine the form of equation (3.5). In this model, it is conceivable that the loop does not represent a fully paired region, i.e. along the loop, there are a certain number of paired and unpaired sections. We suppose that the number of paired sections within the loop may not adjust to reach true thermodynamic equilibrium over the duration of the experiments and remains fixed. This may be true when there are sufficiently large activation barriers for creating and destroying paired sections. Now, if one tries to reduce *L*_loop_, the sizes of both the paired and unpaired regions inside the loop diminish. Therefore, if the free energy of the sections scales nonlinearly with their size, in this quasi-equilibrium state, we can have *F*_loop_(*L*_loop_) nonlinear in *L*_loop_. For instance, for the unpaired sections, this nonlinear dependence could be attributed to unfavourable electrostatic repulsion. For a conserved number of unpaired sections, the decrease in their size will lead to closer proximity of the segments making up the unpaired sections, or increase in their bending energy. In Discussion, we consider further effects underpinning the nonlinear behaviour of the unpaired and paired sections. Now, we formally study the consequences of this phenomenological model.

**Figure 3.**
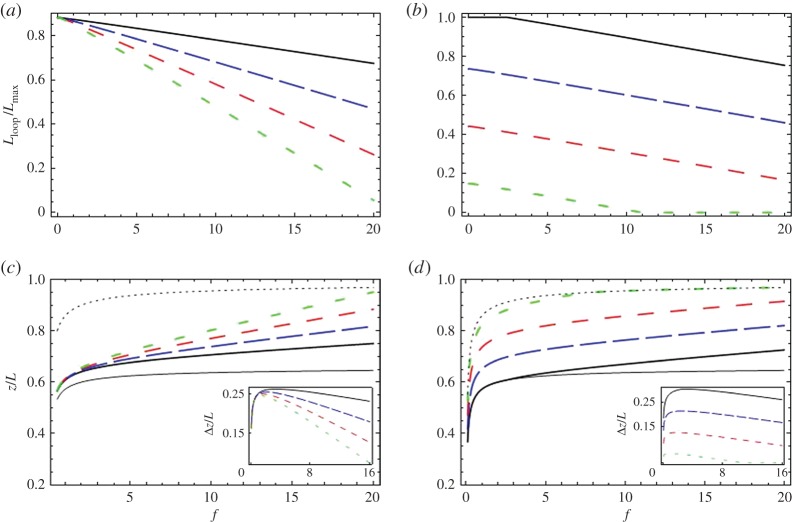
Exploring the effects predicted by model 2. We show, as in figure 2, plots of *L*_loop_ and *z* for various values of model parameters, calculated using equations (3.1), (3.2) and (3.5). Again, a value of *l*_p_=500 Å is used (in equation (3.2)). Plots of *L*_loop_, normalized over $L_{\max}\approx 34\;000$ Å (the maximum length for pairing of the homologous segments), are shown in (*a*,*b*). Plots of *z* divided by the total DNA length *L* are shown in (*c*) and (*d*). As in figure 2, the thin solid line is the extension-force curve expected for total homologous pairing ($L_{\mathrm{loop}}=L_{\max}$) and the thin dotted line is that for no pairing (*L*_loop_= 0). The insets in (*c*,*d*) show Δ*z*—the difference in extension curves with no pairing at all (thin dotted line) and those generated with homologous pairing. In (*a*,*c*), *L*_0_=30 000 Å and the thick solid, long dashed, medium dashed and short dashed lines correspond to the values of *β*=15 000, 30 000, 45 000, 60 000 Å^2^/*k*_B_*T*. In (*b*,*d*), *β*= 20 000 Å^2^/*k*_B_*T* and the solid, long dashed, medium dashed and short dashed lines correspond to the values *L*_0_=35 000, 25 000, 15 000, 5000 Å(we considered even one value of *L*_0_ larger than $L_{\max}$ but, as *L*_loop_ can never exceed $L_{\max}$, in (*b*) the magnitude of *L*_loop_ is limited to $L_{\max})$. (Online version in colour.)

Minimization of equation (3.5), combined with equation (3.1), with respect to *L*_loop_ is simple and leads to an expression for *L*_loop_ presented in the electronic supplementary material. This model describes a smooth continuous transition from *L*_loop_=*L*_0_ to *L*_loop_=0, as we increase the pulling force. In figure 3, we plot both *L*_loop_ and *z* for various values of the model parameters.

Increasing *β* increases the rate at which *L*_loop_ diminishes with increasing force, while increasing *L*_0_ shifts *L*_loop_ up by roughly a force-independent amount. Again, *L*_loop_ cannot exceed the total homology length $L_{\max}$. In addition, it stays at *L*_loop_=0 once there is no pairing. The difference in extension between the control and a construct, Δ*z*, calculated in this model has the same trend with increasing force as in Model 1. We will fit Δ*z* calculated from both Models 1 and 2 to the experimental data.

### (f) Fitting experimental data

We fitted the extensions of the λ-phage dsDNA controls, *z*_λ_, using the WLC extension formula (equation (3.3) with *L*_loop_=0), thus obtaining a value of the bending persistence length *l*_p_ for each salt concentration. Note that the fit values for *l*_p_ lie close to that commonly assumed for dsDNA. This indicates that we in fact deal with a single molecule in the pulling experiments, as assumed above. A typical fit is shown in S1 of the electronic supplementary material, figure S2.

Although we performed some experiments with a 60 kb control molecule constructed by adding 10 kb of heterologous dsDNA to the end of a λ-phage molecule, most of our control experiments simply used a λ-phage dsDNA molecule. Given that the constructs were 6/5 times longer than the majority of λ-phage controls, we had to rescale the control extension curves by this factor. Since *z* scales linearly with *L*, when there is no looping, such a procedure is justified. We then subtracted from the rescaled fitted extension values those for the construct at each given pulling force. Thus, we obtainedΔ*z*=(6/5)*z*_λ_−*z*_c_, where *z*_c_ are extension values for the constructs.

**Figure 4.**
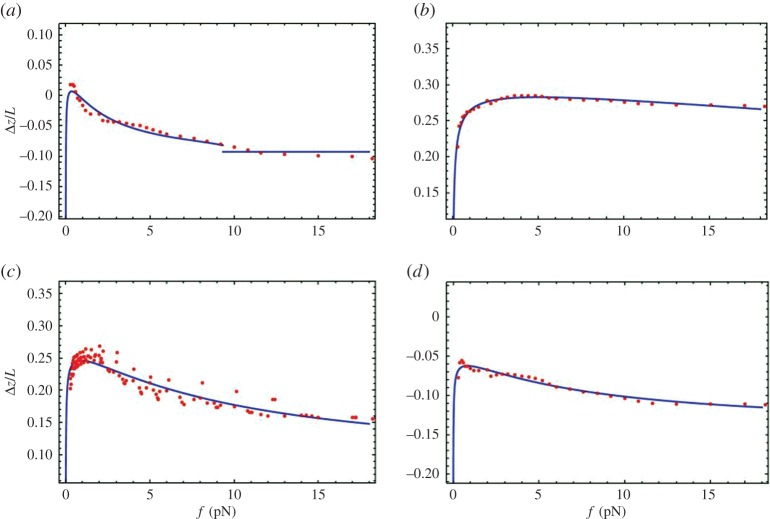
Fits of Model 1 to experimental extension–force data. These are shown for one representative experimental run at 24°C (each panel displays the results for a particular concentration of NaCl). The experimental data for Δ*z*/*L* versus pulling force *f* are shown as points. The curves (theory) are generated by minimizing equations (3.1) and (3.4). Note that for some of the plots the values are negative due to ${\tilde{x}}_{\mathrm{est}}$ being negative, where the construct experiment has a larger offset. The salt concentrations are: (*a*) 0.15 M, (*b*) 0.5 M, (*c*) 1 M and (*d*) 3 M. The fit values *r*=*a*/*ε*_loop_ and *ε*_loop_, obtained for each experimental run, as well as average values for the range of temperatures and ionic conditions considered in this study, are given in the electronic supplementary material, S5. (Online version in colour.)

**Figure 5.**
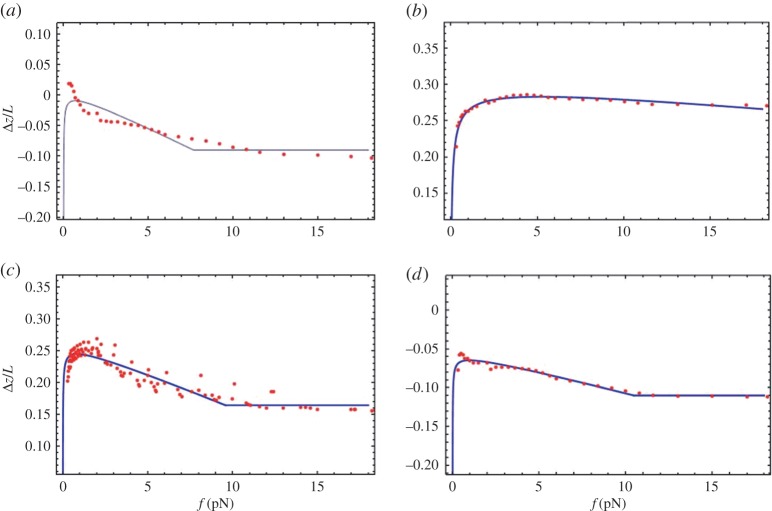
Fits of Model 2 to experimental extension–force data. The same as in figure 4, but with the curves generated by minimizing equations (3.1) and (3.5). The fit values, of *L*_0_ and *β*, for each experimental run, as well as average values for each temperature and ionic condition considered, are given in the electronic supplementary material, S5. (Online version in colour.)

The most important result of the analysis is that Δ*z* is not zero. Crucially, there is a strong reason to believe that it is not an artefact of an experimental error, because all values of Δ*z* show a clear universal trend with increasing pulling force (figures 4 and 5), qualitatively similar to theoretical results (figures 2 and 3). Finite Δ*z* cannot be accounted for uncertainties in the bending persistence length (shown in figure 1 and electronic supplementary material, S1); otherwise, we could not have obtained such a universal trend. In addition, the persistence length of the construct should be the same as that of λ-phage dsDNA, as we consider long genomic DNA where sequence dependences are washed out over large sequence tracts.

We fitted these Δ*z* data to

3.6 $$\Delta z=\left(1-\sqrt{\frac{k_{B}T}{4l_{p}f}}\right)2L_{\mathrm{loop}}(\;f)+{\tilde{x}}_{\mathrm{est}},$$

where the force dependence of *L*_loop_( *f*) is either calculated using Model 1 or 2.

The algorithm that calculates the separation between the centre of the magnetic bead and the capillary edge produces consistent values for a given single-molecule experiment. However, there is a constant offset between the actual dsDNA extension and the algorithmically determined separation between the surface of the bead and the capillary surface. Different single molecules have slightly different offsets. Thus, in equation (3.6), we include a force-independent offset, ${\tilde{x}}_{\mathrm{est}}$. Regardless of the model, this constant simply represents a vertical shift of the curves. Although it is to be determined by fitting, it has no physical significance for the interpretation of the model. It, thus, should not be considered as a fitting parameter.

We first show (figure 4) the fits to the data for Δ*z*, using equation (3.4), for one representative run for each salt concentration. Generally, we fitted the data of all experimental runs, and agreement was generally very good; all curves did indeed show the same trend. In the results presented here, to minimize the number of fitting parameters, *b* was chosen so that *b*=2*l*_p_ for all fitted curves. Here, *l*_p_ was fitted to the control data obtained at the same temperature and salt concentration, and thus it is not as an independent fitting parameter for Δ*z*. For non-zero Δ*z* regions, the shape of the curves is relatively insensitive to a variation of *b*. We see in fitting the data of figure 4*a* a small discontinuity in the theoretical curve (not resolved in the experimental data), due to the factor exp(−*b*/*L*_loop_) destabilizing the energy. One could have got rid of this discontinuity by choosing a smaller value of *b* than the Kuhn length, 2*l*_p_, but this does not seem physically reasonable (when considering dsDNA as a freely jointed chain, the Kuhn length is the effective length of each of the segments that are assumed rigid). Not counting the offset, ${\tilde{x}}_{\mathrm{est}}$, the fitting parameters are *ε*_loop_ and the ratio, *r*=*a*/*ε*_loop_. Their values, obtained for different experimental runs performed at various salt concentrations, are displayed in the electronic supplementary material, S5. For *ε*_loop_, due to a large uncertainty in the fitted values (cf. electronic supplementary material, figure S5) from different runs at the same conditions, it is difficult to infer any trend with varying salt concentration and temperature.

Next, we fitted the data for Δ*z* using equation (3.5). The representative fits (one experimental run for each salt concentration) are shown in figure 5 for various salt concentrations. These fits, for all experimental runs, are also reasonable. The values of the fitting parameters *β* and *L*_0_ for various salt concentrations are presented in the electronic supplementary material, S5 for temperatures 24°C, 37°C and 40°C. Again, in fitting model 2, due to large uncertainty in the fitted values (cf. electronic supplementary material, figure S6.) from different runs, we could not reliably discern trends with changing salt concentration or temperature.

## 4. Discussion

We have demonstrated clear difference between the extension values for the construct containing the head to tail repeated 10 kb sequence and controls that do not contain any self-homologous tracts longer than a few base pairs. The extension difference between the construct and the controls indicates the existence of homology-dependent looping between the long identical dsDNA segments. Though the size of the extension difference varied with buffer conditions and temperature, dsDNA containing the homologous 10 kb regions always showed significantly shorter extension than the controls at forces approximately 1–5 pN, with many buffer conditions showing a deviation over a wider range of forces. This is evidence in favour of a favourable interaction between homologous segments. We will begin by discussing qualitative features of the homology-dependent looping, but later in the discussion, we will consider possible mechanisms that could produce the observed behaviour.

Theories which assume that the binding energy is a linear function of the paired length suggest that the length of fluctuations should increase strongly as the force approaches a critical value and that the loop should abruptly vanish above the critical force [42]. By contrast, in our experiments, the fluctuations do not change significantly as the applied force increases. In addition, the extension difference between the construct and control, Δ*z*, changes gradually at low forces: it often first increases and then decreases, as is illustrated in figures 4 and 5. The initial increase is attributable to the force-dependent factor that sits in front of *L*_loop_ in equation (3.6); this is due to WLC behaviour of the un-looped part of the DNA. The physical explanation why Δ*z* decreases with decreasing force, at sufficiently low force values, is due to the fact that the non-homologous sections of the DNA start to coil (for both constructs and controls); as well the paired region. Thus, both extensions of both constructs and controls will go down as such sections only contribute to the extension. This overall effect is represented by the WLC factor, in equation (3.3), which may cause reduction in Δ*z*. Indeed, at zero pulling force, there is loss of directionality in the end-to-end distance of DNA molecules, due to fluctuations in the WLC. At this point, the extension is effectively zero, in all cases; and thus, here, we would expect Δ*z* should be zero too. Therefore, as one goes to sufficiently low enough forces, there should always be a reduction in Δ*z*, regardless of the change in *L*_loop_. However, in some of the experimental data monotonic behaviour in Δ*z* is seen (cf. figures 1 and 4*a*); but still a reduction in Δ*z*, with reduced pulling force, is expected to happen at lower pulling forces. In some of these cases, this non-monotonic behaviour may lie at forces below those for which equation (3.3) is valid, as well as the force range of the presented experimental data. At larger force values, the decrease in Δ*z* can be explained by a monotonically decreasing *L*_loop_; there is no evidence for an abrupt change in *L*_loop_. As argued in a previous section, this requires the free energy function to depend nonlinearly on the length of DNA. In trying to explain this monotonic change in *L*_loop_ at low forces, we have generated two types of models.

In the first model, for positive values of *ε*_loop_ in equation (3.4), the second term with a positive value of parameter *a* stabilizes the loop against unlooping, which effectively accounts for interaction between different homologously paired sections of the loop. In this case, pairing of each homologous section is metastable. Owing to fluctuations, the overall interaction between the long homologous regions should be considered as dynamically changing. The fluctuations take place both at the level of pairing of homologous segments and of interactions between paired sections. Corresponding conformational changes might be slow due to significant energy barriers. When homologous regions pair or homologously paired regions interact, there could be a drawing-in of counterions into the space between the homologous segments. This reduces the repulsion between them and further promotes pairing. In addition, in such process, ordered water near the dsDNA surfaces may be released into the bulk increasing the entropy. Again, these processes may be transient. However, the entire loop might be stable with parts of dsDNA coming together and apart, when one considers the sum of the mean potential energy and entropy for this state.

This model fits the data reasonably well, with *ε*_loop_>0. Positive values of *ε*_loop_ suggest that we do have the partial looping at zero pulling force, discussed in the theory section. However, this first model is not without its problems. Such a model might be more suitable *for low forces*, as the values of the fitting parameters are a bit extreme for the model to be appropriate for the case of large forces. First, in fitting the data, we chose for *b* the value of the Kuhn length, 2*l*_*b*_; its value could not be smaller. This value of *b* was chosen not to induce noticeable sudden transitions to *L*_loop_=0, unseen in the experimental data. In addition, large values of *a* are needed to fit the data (a typical value of *a* is 100 *k*_B_*T*/ Å^1/2^). This suggests that this additional stabilizing interaction is very strong, which also might not be that realistic. Thus, we considered also another model.

In a more phenomenological Model 2, we suppose that nonlinearity comes from a quasi-equilibrium state. Along the loop, we suppose that we have sections of paired and unpaired homologous segments (loop bubbles). If the number of paired sections is fixed, when the pulling force changes, the system is assumed not to be in true equilibrium. That is to say, as the molecule is pulled, paired regions do not coalesce or are pulled completely apart to diminish the free energy. Indeed, this situation supposes a large kinetic barrier to prevent changes in the number of paired regions.

As the length of a pairing region increases, the two helices will start to fall out of alignment with each other; i.e. the relative azimuthal orientation between the two minor grooves will change as one moves along the paired region [23,45]. One reason for this misalignment is due to thermal twisting fluctuations that distort the shape of the two homologously paired helices differently [23]. A second reason is due to bending fluctuations in which the centre line of the loop undulates. This causes misalignment because, in a bend of loop centre line, one helical molecule may have to use up more contour length, and so more helical pitches, than the other one. This misalignment may be aggravated by intrinsic structural distortions away from an ideal helix structure [29] present in DNA due to imperfect stacking of the base pairs [27,28]. Therefore, if the helical structure is responsible for the pairing interaction, then as one increases the length of the paired regions, the attractive forces will be weakened due to the increasing mismatch between helices [30,46]. The upshot is that the reduction in free energy due to pairing may not scale linearly with the size of the pairing region. Loop bubbles help to restore the match and bring down the free energy by increasing the number of paired regions, but decreasing their size.

There is another possibility for the nonlinear behaviour of the pairing regions, if pairing between identical sequences depends locally on the base-pair sequence (discussed below). Then, for optimal pairing to occur, the number of base pairs per turn should be an integer, instead of an average value of 10.5 for an isolated DNA molecule in solution. To make this adjustment, the torsional strain may get kinetically trapped in a non-uniform distribution, accumulating with the increasing length of the paired section. A similar idea has been explored in a study of the effect of homology on the energetics of the formation of the ssDNA–RecA–dsDNA complex in homologous recombination [47].

Additional nonlinearity may come from the loop bubbles. In those regions, the two helices are considered to be further apart to increase the entropy of the loop. The nonlinearity possibly arises, here, from longer range electrostatic repulsion, which may become more pronounced with the decreasing size of the loop bubbles as the two segments would be on average closer to each other. The nonlinearity in the response of these regions, as they are made smaller, may also come from a reduction of entropy, as loop bubbles are made smaller, as well as the bending energy required to separate the two helices to reduce the electrostatic repulsion between them. To model all these factors, in equation (3.5), we chose a quadratic form as the simplest possible expression for the nonlinearity.

In fitting the data, Model 2 does reasonably well. Similar to Model 1, the fits suggest partial looping at zero pulling force, as the length of the identical sequences forming the loop, *L*_0_ is found to be less than their total length, $L_{\max}\approx 34\;000$ Å. However, this model, unlike the first one, does not give any reason why this may be so.

One clear advantage of model 2 is its simplicity. It can be used empirically, regardless of the mechanism that causes the smooth change in *L*_loop_, which the experimental data clearly points to. It has two useful parameters that quantify the extent of looping at low forces (*L*_0_), and the rate of unlooping (*β*) as pulling force is increased.

A combination of the two models might be in place: at low forces, when the loop is sufficiently long, the first model is most appropriate, but as the pulling force is increased, the second model may be more suitable. The fact that we could not unambiguously discriminate between the two models leaves room for further investigations and design of new experiments. We, of course, cannot exclude the possibility of any other model, if justified and better in fitting experimental data.

Both models are not specifically linked to underlying microscopic mechanisms of the homologous pairing, although one can speculate what these are. Here, we discuss two distinct types of preferential pairing mechanisms between identical sequences, both relying on the helical structure of dsDNA.

The first one, which was initially hypothesized by Kornyshev & Leikin [30], relies on the fact that the distortions in the helix structure depend on base-pair sequence [27–29]. Here, the actual microscopic details of the interactions do not matter, nor need they be sequence specific; simply, they only need to involve a proper account of the distorted helical symmetry of the molecules. The initial calculation of the recognition energy [30] and the recognition well as a function of sliding one homologous segment along the other [32,33] were done using mean-field electrostatics [23]. In this mechanism, it is sequence-dependent similarities in the patterns of distortions of the helical structure of the molecules that initiate pairing. Indeed, it was suggested by Kornyshev & Leikin [30] that two identical (homologous) sequences in one-to-one juxtaposition would have lower interaction energy than those which were completely different or for homologues shifted along each other (for review, [23,48]).

A second mechanism is that homologous pairing depends microscopically on specific base-pair content. Recent experiments *in vivo* have suggested that this mechanism might be important [49]. For such an interaction, we can suggest one possible candidate. This relies on new simulation results [22] indicating that monovalent ions may, in fact, be more localized in the minor grooves of AT sequences and major grooves of GC sequences than was originally expected. This localization is due to preferential interactions with the base pairs of the two molecules. Thus, one can envisage a situation where the identical segments may pair through (i) the formation of cages formed by the facing minor grooves of the two molecules and the counterions, preferentially at AT bases and (ii) those formed by counterions preferentially localized within facing major grooves, preferentially at GC bases. There is, in simulations for poly (AT) DNA, an indication that the minor grooves facing each other and counterions lying in between may facilitate weak attraction [34]. Such cages, if formed, may lower the interaction energy for identical sequences, as AT (GC) sequences will want to align with AT (GC) sequences on the other molecule, with the two minor (major) grooves facing each other. In this *local* homology-dependent interaction mechanism, the similarities (or dissimilarities) in the patterns of helix distortions will again play a role, as the base pairs will need to align correctly for such interactions to be sufficiently strong. We might be able to distinguish between the local and global pairing mechanisms in the future, by designing special constructs originally considered *in vivo* [49].

One other question is why pairing between identical dsDNA sequences, in the absence of DNA condensing ions, does not cause their spontaneous aggregation. For homologous short fragments, mild osmotic stress needs to be applied for them to aggregate [6]. However, in this case, the entropy gain from each small fragment being free in solution, as opposed to being in an aggregate, is likely to be much larger than for unlooping of the two long identical segments attached to each other. In addition, braiding may further stabilize the pairing of two long identical segments [50]. Recent work that models the initial interaction as the pairing between rigid rods (regions of dsDNA that are shorter than the persistence length) indicates that the homology-dependent torque required to angularly align the two rods would prevent aggregation unless long regions of homology are present; however, more needs to be understood, here.

## 5. Conclusion

The hypothesis that homologous regions of chromosomes can pair without any assistance from proteins has been tested in this work through magnetic bead-assisted single-molecule force–extension experiments. We have found that the specially constructed 40–60 kb long dsDNA with two 10 kb homologous sections running head-to-head, in opposite direction will *fold on itself* under physiologically reasonable conditions.

We compared the force-dependent extension for molecules containing homologous fragments and those which do not, of the same contour length. We clearly see differences in the extension curves. These signatures of the folding of DNA containing homologous sequences persist even above the pulling force of 10 pN.

In these experiments, we observed homology-dependent pairing between dsDNA molecules in real time and measured the dependence of this pairing on an applied force that attempts to pull the paired regions apart.

The continuous character of the force-induced unfolding (no abrupt change with force) is consistent with the dynamic character of the pairing of homologous regions. Such interaction may allow homologous regions of chromosomes to rapidly find each other in the genetic haystack. Once such protein-independent interaction weakly pairs homologous regions, additional interactions between the paired chromosomes could be provided by proteins. An important criterion is that the protein-independent interaction alone does not irreversibly weld homologous chromosomes together.

The experimental data are compatible with two phenomenological models. The first one describes metastable pairing stabilized by collective attractive interactions between homologously paired regions, the form of which is deduced by simple polymer scaling arguments. The second model describes a quasi-equilibrium situation, where the number of paired regions and loop bubbles stays constant although they shrink with a nonlinear spring like response under the pulling force.

This is a fourth independent experimental demonstration of the existence of such interaction, achieved *in vitro* (in a test tube). It is new evidence that homologous regions of chromosomes have an option to recognize each other as a result of properties intrinsic to dsDNA.

## Supplementary Material

Supporting Material

## Data accessibility

Data supporting this article are included in the electronic supplementary material. S1 contains additional supporting experimental data; S2 contains details on the background of Model 1; S3 discusses the approximations behind the equations for *L*_loop_; S4 presents plots of the free energy for model 1 as well as a plot showing how *L*_loop_ varies with the parameter *b* in that model; S5 shows the fitted the values of the model parameters; S6 specifies exactly the DNA text that was added to the end of the λ DNA in the constructs.
