# Supplementary material for: Evidence of protein-free homology recognition in magnetic bead force–extension experiments
Source: Proc Math Phys Eng Sci. 2016 Jul;472(2191):20160186. doi: 10.1098/rspa.2016.0186 (PMC4971244; doi:10.1098/rspa.2016.0186)
Supplement: Supporting Material [file rspa20160186supp1.pdf]

Supporting Material

S.1. Additional experimental data

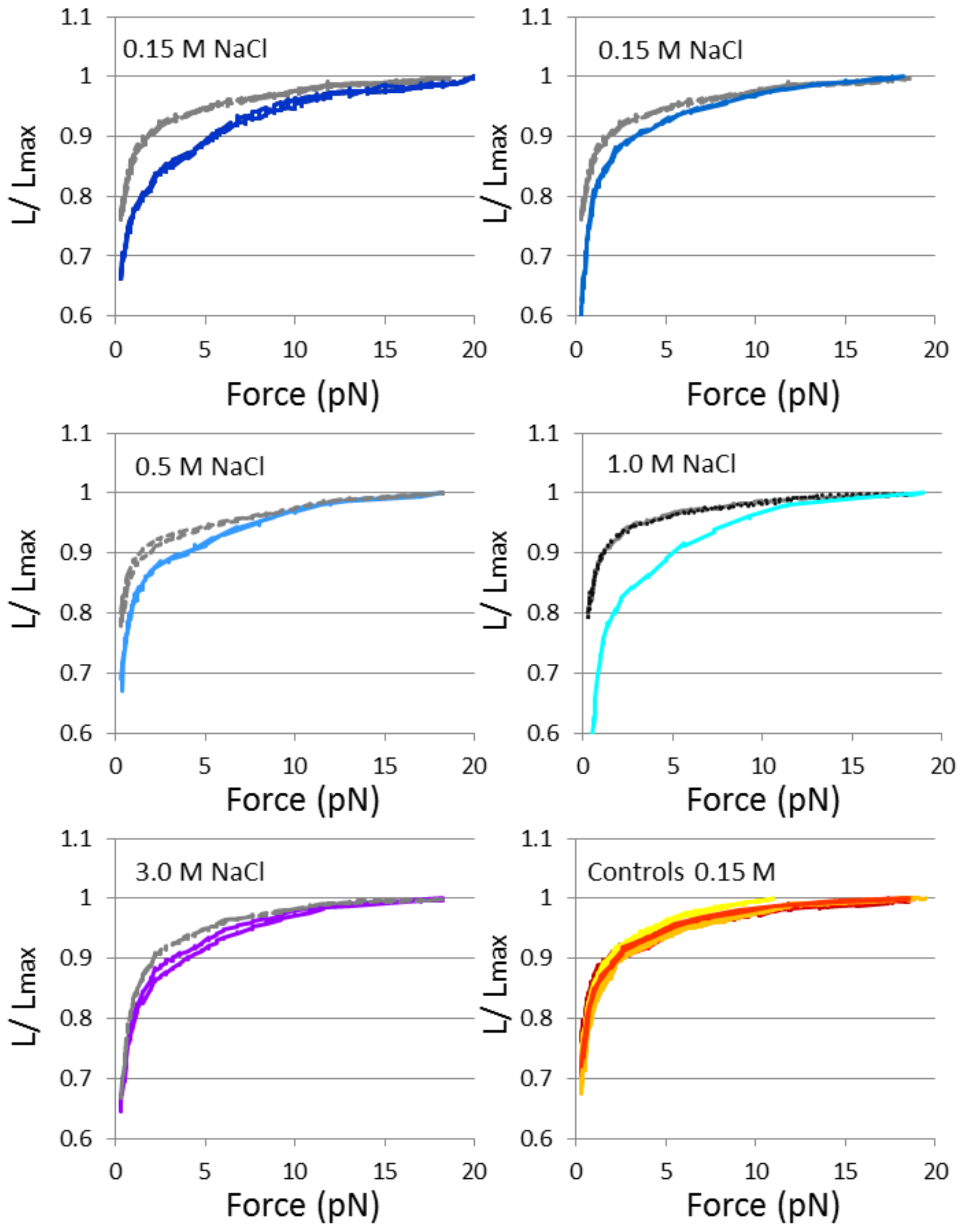

Fig. S.1. Additional representative results showing the extension vs. force curves (increasing and decreasing force) in 0.15, 0.5, 1.0, and 3 M NaCl and 50 mM phosphate buffer at 24°C for a dsDNA construct (coloured curves) and a control curve for dsDNA without homologous regions in the corresponding ionic conditions (dashed gray with an additional black points for a second control experiment). Five control curves obtained with different beads at 24°C and phosphate buffer- 0.15 M NaCl are shown in yellow, orange, light red, red, and dark red (bottom right).

First, we present unprocessed experimental data (at 24°C) for other experimental runs in addition to what is shown in Fig.1 of the main text. In the top two panels we present the results of two separate controls and construct experiments done at the same conditions. We see that the difference between the construct and control experiments is present in both experiments. Though, its magnitude varies somewhat, leading to a large uncertainty in the fitting parameters as seen below.

We also show that this difference persists with changing salt concentrations between 0.15 M and 3M. Last of all, we show that the variation in the curves of multiple control experiments is much smaller than the differences seen between construct and control curves at a given salt concentration. This is first seen by looking at the middle right panel where we plot two control curves against one construct; clearly the biggest difference is between the construct and the two controls that lie close to each other.

Furthermore, we show in the bottom right hand panel the curves of multiple control experiments for 0.15 M NaCl, again the difference is much smaller than observed differences between constructs and controls.

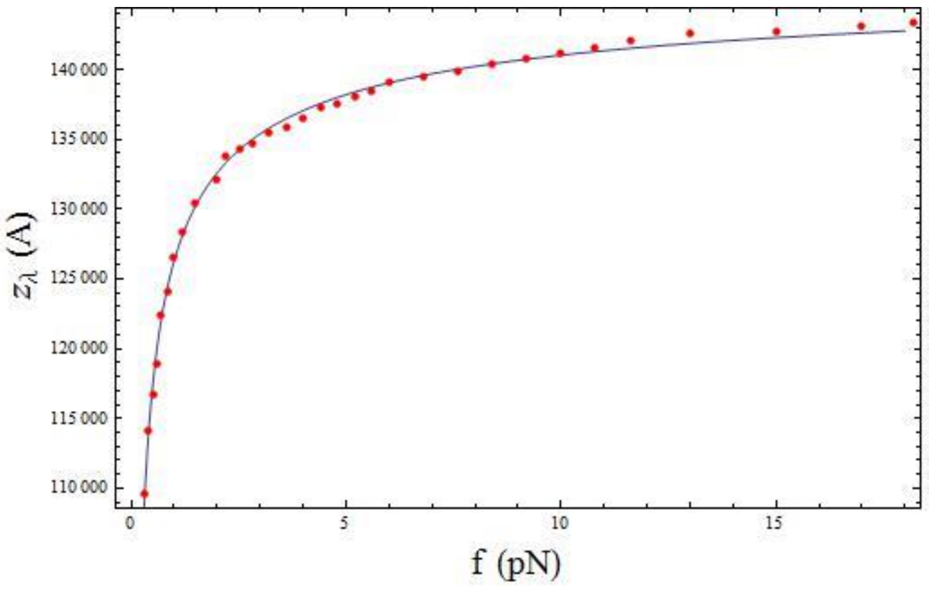

Fig. S.2. WLC fit to the control experiment. In this figure we show a fit to the data for the control DNA in 0.15M NaCl aqueous solution to the worm like chain extension formula. Using Eq. (3) of the main text with $L_{loop} = 0$, the fitted value of bending persistence length is $l_p = 636.57$ Å.

In Fig. S.2 we show a typical fit of the bending persistence length to control experimental data. Fits were done on control experiment runs and those values were used in fitting Δz. Though the fit is quite good, the value of $l_p = 636.57\text{Å}$ should really be considered as an apparent value bending persistence length as its value is larger than the commonly accepted value of $l_p \approx 500\text{Å}$. This value is due to the fit being performed at relatively large values of the pulling force. In fact, there is quite a large spread of values in l_p about $l_p \approx 500\text{Å}$, when we fitted them to available experimental data.

This is because the uncertainty in the extension (for repeated runs) for larger force values affects l_p more than extensions at lower forces, due to the non-linear nature of the extension formula. A fit containing more low force values would likely give a range of values closer to $500\,\text{Å}$. Importantly, we demonstrate that difference between construct and control lies outside this uncertainty in extension values (see Fig S.1).

Fitting extension curves over the range of force values considered reflects, however, better the true nature of these curves and the uncertainty in extension values. One could introduce DNA extensibility into the wormlike chain formula, which would be a small correction at these force values, but this seems rather pointless as the fits to the control curves are adequate.

S.2. Model 1: formulating the non-linear term

We will use polymer scaling arguments to deduce what the form of the non-linear term in the free energy of the loop, supposing that a non-linear term comes from different parts of the loop interacting in an attractive manner. We may write down for the average interaction attractive energy

$$E_{\mathrm{int}} = -\iint_{\Gamma} d\mathbf{r}\, d\mathbf{r}' \rho(\mathbf{r}) V(\mathbf{r}-\mathbf{r}') \rho(\mathbf{r}'). \quad \text{(S.1)}$$

Here, $\rho(\mathbf{r})$ is the local looped DNA density at position $\mathbf{r}$ and $-V(\mathbf{r}-\mathbf{r}')$ is an attractive interaction energy between two parts of the loop at positions $\mathbf{r}$ and $\mathbf{r}'$. Both volume integrals are taken over average volume of space, Γ, that the loop occupies. If we suppose that the interaction is short ranged such that $V(\mathbf{r}-\mathbf{r}') \approx V_0 \delta(\mathbf{r}-\mathbf{r}')$ and we assume the density $\rho(\mathbf{r})$ to be uniform in the volume taken up by the loop, i.e. $\rho(\mathbf{r}) = \bar{\rho}$, then

$$E_{\mathrm{int}} \approx -\bar{\rho}^2 V_0 \Gamma. \quad \text{(S.2)}$$

Now since the density should go as $\rho \propto L_{loop} / \Gamma$ we have that

$$E_{\mathrm{int}} \sim L_{loop}^2 \Gamma^{-1}. \quad \text{(S.3)}$$

We now use polymer scaling arguments to deduce the dependence of E_{int} on L_{loop}. Supposing that volume taken up by the loop goes as $\Gamma \sim R_g^3 \sim L_{loop}^{3\nu}$, so that we have

$$E_{\mathrm{int}} \sim L_{loop}^{2-3\nu}. \quad \text{(S.4)}$$

We will choose the value $\nu = 1/2$. We cannot assert that this value is exact; as for excluded volume polymer it is known to be close to 3/5 in the absence of sufficiently strong attraction between the polymer segments [M.Doi, *Introduction to polymer physics,* Clarendon Press, Oxford, 1996]. In our rather complex system there may well be a balance of the attractions between different parts of the loop and electrostatic repulsions, allowing for a lower value of ν. For simple estimates we take the

value of ν of a simple random walk, thus behaving as a polymer in a 'θ-solvent', which fits the experimental data well. Thus when the loop is large we suggest the form

$$E_{\text{int}} = -aL_{loop}^{1/2}. \tag{S.5}$$

An additional factor $\exp\left(-b / L_{loop}\right)$ is introduced to take account of the bending free energy cost to form a loop that hinders these interactions. This yields the form for F_{loop} presented in Eq. (4) of the main text.

S.3. Equations for the length of the loop

For Model 1, the length of the loop, obtained by numerical minimization of Eq. (4) of the main text, can be well approximated by

$$L_{loop} = \left(\frac{a}{\varepsilon_{loop} - 2g_{WLC}(f)} \right)^2 H\left(\left(\frac{\varepsilon_{loop} - 2g_{WLC}(f)}{a} \right)^2 b \right), \tag{S.6}$$

where

$$H\left(\tilde{b}\right) = \left(0.249 + 1.85\tilde{b} - 12.50\tilde{b}^2 + 46.35\tilde{b}^3 - 76.82\tilde{b}^4\right)\theta(0.315 - \tilde{b}). \tag{S.7}$$

The value of the argument of the function H, $\tilde{b} = (\varepsilon_{loop} - 2g_{WLC}(f))^2 b / a^2$, increases as one increases both b and the pulling force f . One should note that when $\tilde{b}$ exceeds the value 0.315 the $L_{loop} \neq 0$ state is no longer stable, the only stable one is then the $L_{loop} = 0$ state. Thus, we have allowed for the $L_{loop} \neq 0$ state to be metastable. The latter assumes that the energy barrier is sufficiently large for the molecule complete unfolding of the loop, i.e. emergence of the $L_{loop} = 0$ state. Hence, thermal fluctuations are not expected to cause a significant unfolding. We plot this function and the resulting expression for z in Fig.2 and use it to fit the experimental data shown in Fig. 5 (both in the main text), with the additional constraint that the maximum value of L_{loop} is $L_{\max} = 34000\text{Å}$.

For Model 2 the minimization is simple and we obtain

$$L_{loop} = L_0 + \beta g_{wlc}(f), \tag{S.8}$$

with the requirement that $L_{\max} \geq L_{loop} \geq 0$ We plot this function (and the resulting expression for z) in Fig.3 and use it to fit experimental data in Fig 6 of the main text.

S.4. Additional parameter plots for Model 1

Here, we show additional plots for the readers to get a better feeling of how Model 1 works. In Fig. S.3 we plot the L_{loop} depending part of the total free energy, $F_{loop}(L_{loop}) - 2g_{wlc}(f)L_{loop}$, *vs* L_{loop}. We see that decreasing a, as well as increasing either $\varepsilon = \varepsilon_{loop} - 2g_{wlc}(f)$ or b destabilizes the free energy minimum at $L_{loop} \neq 0$.

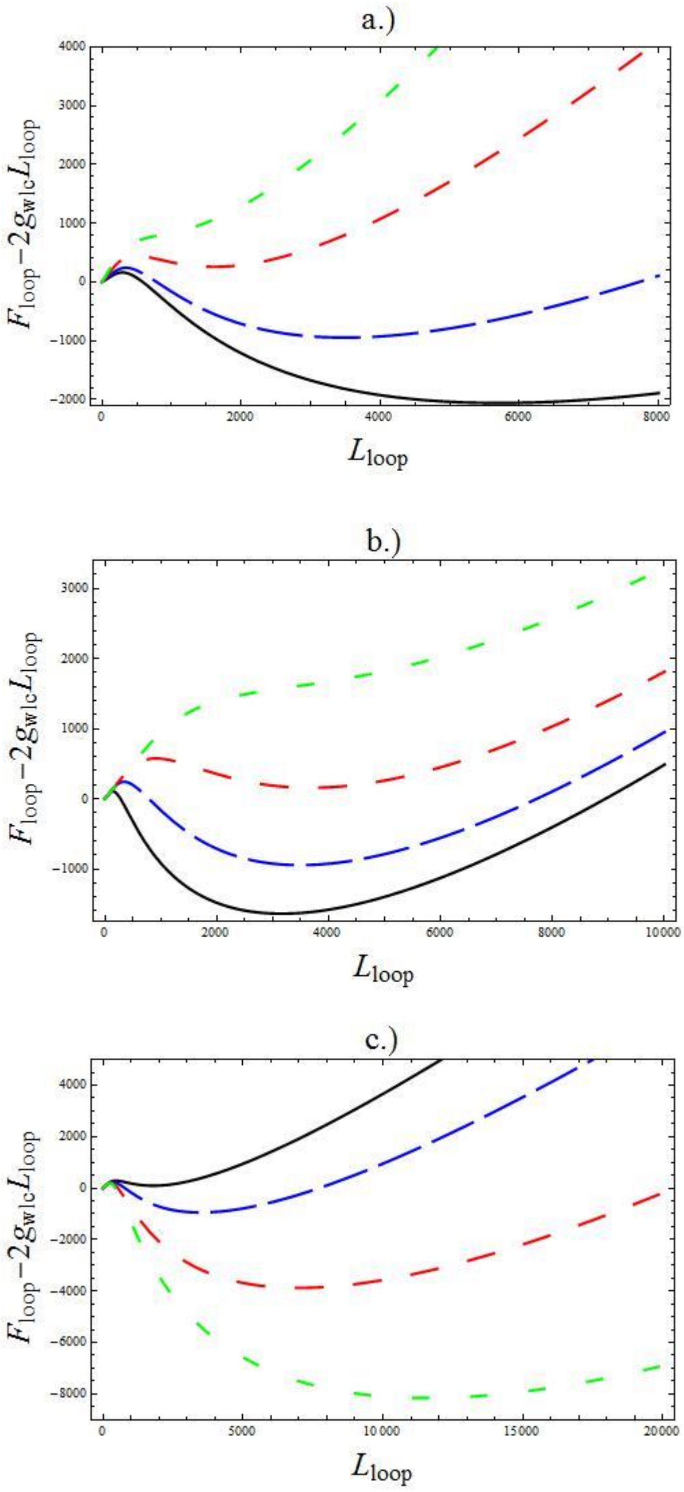

Fig. S.3. Plots of the free energy contribution $F_{loop}(L_{loop}) - 2g_{wlc}(f)L_{loop}$ for various values of the parameters a, b and $\varepsilon = \varepsilon_{loop} - 2g_{wlc}(f)$.

a.) We fix $b = 1000\text{Å}$ and $a = 100 k_B T / \text{Å}^{1/2}$ The thick solid (black), long dashed (blue), medium dashed (red), and short dashed (green) lines correspond, respectively, to the values $\varepsilon = 0.75,\ 1,\ 1.5,\ 2\ k_B T / \text{Å}$

b.) We fix $a = 100 k_B T / \text{Å}^{1/2}$ and $\varepsilon = 1 k_B T / \text{Å}$. The thick solid (black), long dashed (blue), medium dashed (red), and short dashed (green) lines correspond to the values $b = 500,\ 1000,\ 2000,\ 4000\ k_B T / \text{Å}$.

c.) We fix $b = 1000\,\text{Å}$ and $\varepsilon = 1 k_B T / \text{Å}$. The thick solid (black), long dashed (blue), medium dashed (red), and short dashed (green) lines correspond to the values $a = 70,\ 100,\ 150,\ 200\ k_B T / \text{Å}^{1/2}$.

In Fig S.4 we show the effect of changing b on the value of $L_{loop} / L_{\max}$. Here we see that the shape of the continuous part of the curves where $L_{loop} \neq 0$ is not affected much by changing b, but increasing b reduces the force at which we see a sudden jump down to $L_{loop} = 0$, as well as the magnitude of that jump.

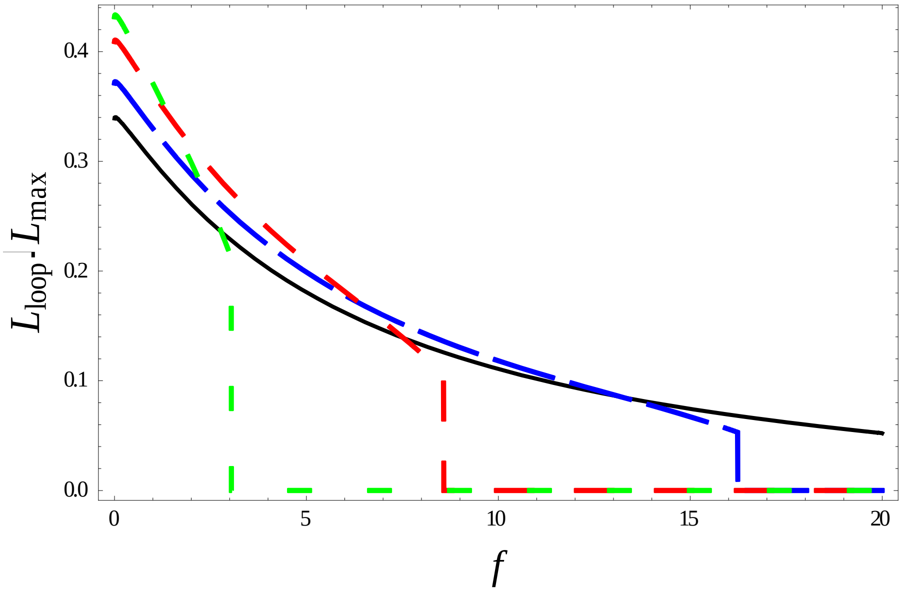

Fig. S.4. The ratio of loop length to maximal extension as a function of the pulling force: the effect of Kuhn length. Here we fix $\varepsilon_{loop} = 0.5 k_B T / \text{Å}$ and $a = 100 k_B T / \text{Å}^{1/2}$, and plot curves for different values of Kuhn length: $b = 1000\,\text{Å}$ (black solid line), $b = 2000\,\text{Å}$ (blue long dashed line), $b = 4000\,\text{Å}$ (red medium dashed line) and $b = 8000\,\text{Å}$ (green short dashed line).

S.5. Model parameter fit values

Here, we present values for model parameters fitted to various experimental runs. Shown in Fig. S.5 and Fig. S.6 are the fitted values of Model 1 and 2, respectively, to the available experimental data. The fitted values, averaged over experimental runs done at the same conditions, are also presented in Tables S.1 and S.2. Some of the data seem to indicate that the degree of pairing first increases with salt concentration, before reducing. However, the data of multiple experimental runs have large scatter; the uncertainty on the fitted values is large, so the existence of this trend remains uncertain.

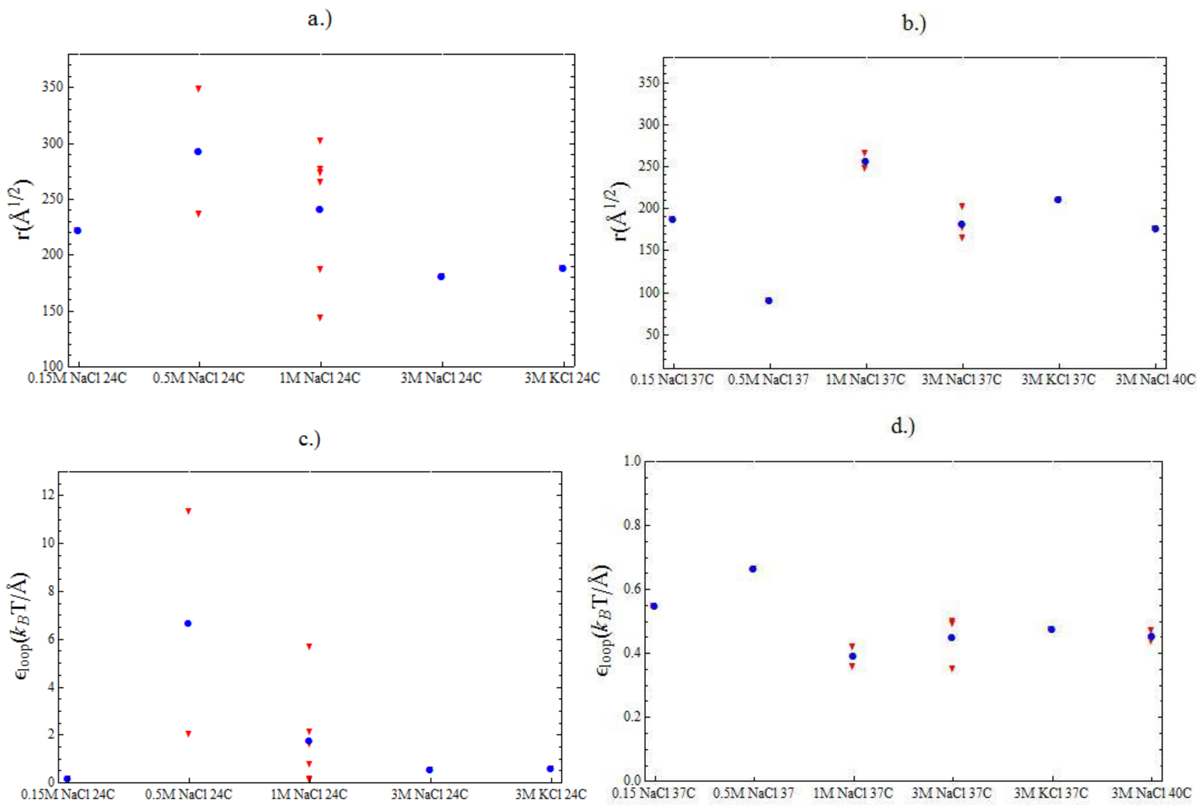

Fig S.5. The fitted parameter values of Model 1, $r\ (= a/\varepsilon_{loop})$ and ε_{loop} , for different temperatures and ionic conditions. Panels a.) and b.) show the fitted values for the ratio r , while c.) and d.) display the values of ε_{loop} . As indicated in the labels on the x-axis, a.) and c.) correspond to data at the lower temperature value 24° C, while b.) and d.) correspond to the higher temperature values of 37° C and 40° C. The red triangles correspond to individual experimental runs, whereas the blue circles correspond to the averaged values.

Temperature	Ionic conditions	Mean Fitted values of $r = a/\varepsilon_{loop}$	Mean Fitted values of ε_{loop}
24°C	0.15M NaCl	222 $Å^{1/2}$	0.167 $k_BT/Å$
24°C	0.5M NaCl	293 $Å^{1/2}$	6.68 $k_BT/Å$
24°C	1M NaCl	241 $Å^{1/2}$	1.75 $k_BT/Å$
24°C	3M NaCl	181 $Å^{1/2}$	0.547 $k_BT/Å$
24°C	3M KCl	188 $Å^{1/2}$	0.571 $k_BT/Å$
37°C	0.15M NaCl	187 $Å^{1/2}$	0.547 $k_BT/Å$
37°C	0.5M NaCl	90.6 $Å^{1/2}$	0.664 $k_BT/Å$
37°C	1M NaCl	256 $Å^{1/2}$	0.390 $k_BT/Å$
37°C	3M NaCl	182 $Å^{1/2}$	0.450 $k_BT/Å$
37°C	3M KCl	210 $Å^{1/2}$	0.476 $k_BT/Å$
40°C	3M NaCl	176 $Å^{1/2}$	0.454 $k_BT/Å$

Table S.1. **The average values of the fitted parameters of Model 1**, $r = a/\varepsilon_{loop}$ and ε_{loop} for the various ionic conditions and temperatures probed in the pulling experiments.

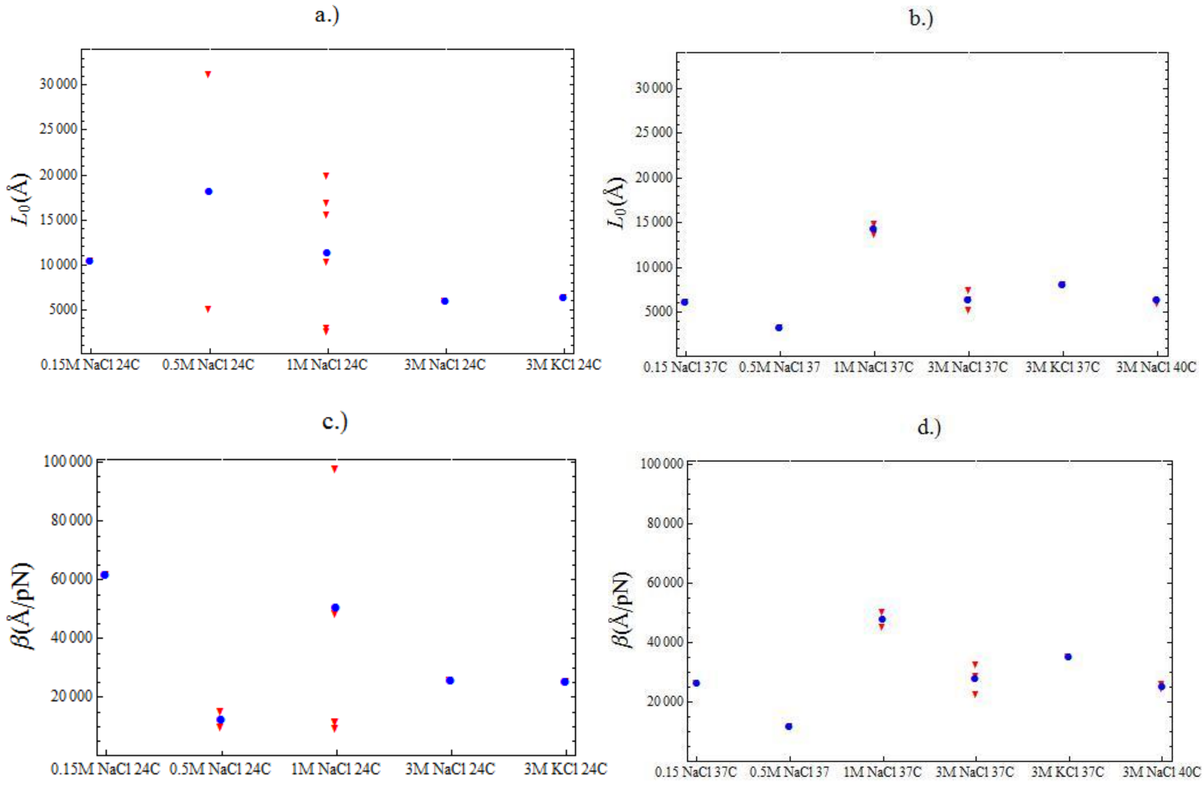

Fig. S.6. The fitted parameter values of Model 2, L_0 and β, for different temperatures and ionic conditions. The fitted values for L_0 are presented in a.) and b.) while c.) and d.) display the values of β. As indicated in the labels on the x-axis, a.) and c.) correspond to data at the lower temperature value $24^\circ\,\mathrm{C}$, while b.) and d.) correspond to the higher temperature values of $37^\circ\,\mathrm{C}$ and $40^\circ\,\mathrm{C}$. The red triangles correspond to individual experimental runs, whereas the blue circles correspond to the averaged values.

Temperature	Ionic conditions	Mean Fitted values of L_0	Mean Fitted values of β
24°C	0.15M NaCl	10500Å	61600 Å/pN
24°C	0.5M NaCl	18100Å	12300 Å/pN
24°C	1M NaCl	11300Å	50600 Å/pN
24°C	3M NaCl	5880Å	25500 Å/pN
24°C	3M KCl	6790Å	23400 Å/pN
37°C	0.15M NaCl	6090Å	26200 Å/pN
37°C	0.5M NaCl	3200Å	11600 Å/pN
37°C	1M NaCl	14800Å	47700 Å/pN
37°C	3M NaCl	6340Å	27900 Å/pN
37°C	3M KCl	8010Å	35100 Å/pN
40°C	3M NaCl	6340Å	25200 Å/pN

Table S.2. The average values of the fitted parameters of Model 2, L_0 and β, for the various ionic conditions and temperatures probed in the pulling experiments.

S.5. The DNA coding of the homologous segments in the construct DNA

The construct molecules were made by adding an additional DNA segment to the Lambda DNA. This segment was made by PCR reaction amplifying between positions 16322 and 26598 on lambda DNA. The segment was attached so that base pair text (the Lambda DNA base pair sequence from positions 16322 to 26598) ran in the opposite direction to that of the Lambda DNA. The sequence between positions 16322 and 26598 reads as:

```
16322  cctggtgtc tgtgggatat gctgacccat ccgcgctacg gcatggggaa acgtcttggt
16381 gcggcggatg tggataaatg ggcgctgtat gtcatcggcc agtactgcga ccagtcagtg
16441 ccggacggct ttggcggcac ggagccgcgc atcacctgta atgcgtacct gaccacacag
16501 cgtaaggcgt gggatgtgct cagcgatttc tgctcggcga tgcgctgtat gccggtatgg
16561 aacgggcaga cgctgacgtt cgtgcaggac cgaccgtcgg ataagacgtg gacctataac
16621 cgcagtaatg tggtgatgcc ggatgatggc gcgccgttcc gctacagctt cagcgccctg
16681 aaggaccgcc ataatgccgt tgaggtgaac tggattgacc cgaacaacgg ctgggagacg
16741 gcgacagagc ttgttgaaga tacgcaggcc attgcccgtt acggtcgtaa tgttacgaag
16801 atggatgcct ttggctgtac cagccggggg caggcacacc gcgccgggct gtggctgatt
16861 aaaacagaac tgctggaaac gcagaccgtg gatttcagcg tcggcgcaga agggcttcgc
16921 catgtaccgg gcgatgttat tgaaatctgc gatgatgact atgccggtat cagcaccggt
16981 ggtcgtgtgc tggcggtgaa cagccagacc cggacgctga cgctcgaccg tgaaatcacg
17041 ctgccatcct ccggtaccgc gctgataagc ctggttgacg gaagtggcaa tccggtcagc
17101 gtggaggttc agtccgtcac cgacggcgtg aaggtaaaag tgagccgtgt tcctgacggt
17161 gttgctgaat acagcgtatg ggagctgaag ctgccgacgc tgcgccagcg actgttccgc
17221 tgcgtgagta tccgtgagaa cgacgacggc acgtatgcca tcaccgccgt gcagcatgtg
17281 ccggaaaaag aggccatcgt ggataacggg gcgcactttg acggcgaaca gagtggcacg
17341 gtgaatggtg tcacgccgcc agcggtgcag cacctgaccg cagaagtcac tgcagacagc
17401 ggggaatatc aggtgctggc gcgatgggac acaccgaagg tggtgaaggg cgtgagtttc
17461 ctgctccgtc tgaccgtaac agcggacgac ggcagtgagc ggctggtcag cacggcccgg
17521 acgacggaaa ccacataccg cttcacgcaa ctggcgctgg ggaactacag gctgacagtc
17581 cgggcggtaa atgcgtgggg gcagcagggc gatccggcgt cggtatcgtt ccggattgcc
17641 gcaccggcag caccgtcgag gattgagctg acgccgggct attttcagat aaccgccacg
17701 ccgcatcttg ccgtttatga cccgacggta cagtttgagt tctggttctc ggaaaagcag
17761 attgcggata tcagacaggt tgaaaccagc acgcgttatc ttggtacggc gctgtactgg
17821 atagccgcca gtatcaatat caaaccgggc catgattatt actttatat ccgcagtgtg
17881 aacaccgttg gcaaatcggc attcgtggag gccgtcggtc gggcgagcga tgatgcggaa
17941 ggttacctgg attttttcaa aggcaagata accgaatccc atctcggcaa ggagctgctg
18001 gaaaaagtcg agctgacgga ggataacgcc agcagactgg aggagttttc gaaagagtgg
18061 aaggatgcca gtgataagtg gaatgccatg tgggctgtca aaattgagca gaccaaagac
18121 ggcaaacatt atgtcgcggg tattggcctc agcatggagg acacggagga aggcaaactg
18181 agccagtttc tggttgccgc caatcgtatc gcatttattg acccggcaaa cgggaatgaa
18241 acgccgatgt ttgtggcgca gggcaaccag atattcatga acgacgtgtt cctgaagcgc
18301 ctgacggccc ccaccattac cagcggcggc aatcctccgg ccttttccct gacaccggac
18361 ggaaagctga ccgctaaaaa tgcggatatc agtggcagtg tgaatgcgaa ctccgggacg
18421 ctcagtaatg tgacgatagc tgaaaactgt acgataaacg gtacgctgag ggcggaaaaa
18481 atcgtcgggg acattgtaaa ggcggcgagc gcggcttttc cgcgccagcg tgaaagcagt
18541 gtggactggc cgtcaggtac ccgtactgtc accgtgaccg atgaccatcc ttttgatcgc
18601 cagatagtgg tgcttccgct gacgtttcgc ggaagtaagc gtactgtcag cggcaggaca
18661 acgtattcga tgtgttatct gaaagtactg atgaacggtg cggtgattta tgatggcgcg
18721 gcgaacgagg cggtacaggt gttctcccgt attgttgaca tgccagcggg tcggggaaac
18781 gtgatcctga cgttcacgct tacgtccaca cggcattcgg cagatattcc gccgtatacg
18841 tttgccagcg atgtgcaggt tatggtgatt aagaaacagg cgctgggcat cagcgtggtc
18901 tgagtgtgtt acagaggttc gtccgggaac gggcgtttta ttataaaaca gtgagaggtg
18961 aacgatgcgt aatgtgtgta ttgccgttgc tgtctttgcc gcacttgcgg tgacagtcac
19021 tccggcccgt gcggaaggtg gacatggtac gtttacggtg ggctattttc aagtgaaacc
19081 gggtacattg ccgtcgttgt cgggcgggga taccggtgtg agtcatctga aagggattaa
19141 cgtgaagtac cgttatgagc tgacggacag tgtgggggtg atggcttccc tggggttcgc
19201 cgcgtcgaaa aagagcagca cagtgatgac cggggaggat acgtttcact atgagagcct
19261 gcgtggacgt tatgtgagcg tgatggccgg accggtttta caaatcagta agcaggtcag
19321 tgcgtacgcc atggccggag tggctcacag tcggtggtcc ggcagtacaa tggattaccg
```

19381 taagacggaa atcactcccg ggtatatgaa agagacgacc actgccaggg acgaaagtgc
19441 aatgcggcat acctcagtgg cgtggagtgc aggtatacag attaatccgg cagcgtccgt
19501 cgttgttgat attgcttatg aaggctccgg cagtggcgac tggcgtactg acggattcat
19561 cgttggggtc ggttataaat tctgattagc caggtaacac agtgttatga cagcccgccg
19621 gaaccggtgg gctttttttgt ggggtgaata tggcagtaaa gatttcagga gtcctgaaag
19681 acggcacagg aaaaccggta cagaactgca ccattcagct gaaagccaga cgtaacagca
19741 ccacggtggt ggtgaacacg gtgggctcag agaatccgga tgaagccggg cgttacagca
19801 tggatgtgga gtacggtcag tacagtgtca tcctgcaggt tgacggtttt ccaccatcgc
19861 acgccgggac catcaccgtg tatgaagatt cacaaccggg gacgctgaat gattttctct
19921 gtgccatgac ggaggatgat gcccggccgg aggtgctgcg tcgtcttgaa ctgatggtgg
19981 aagaggtggc gcgtaacgcg tccgtggtgg cacagagtac ggcagacgcg aagaaatcag
20041 ccggcgatgc cagtgcatca gctgctcagg tcgcggccct tgtgactgat gcaactgact
20101 cagcacgcgc cgccagcacg tccgccggac aggctgcatc gtcagctcag gaagcgtcct
20161 ccggcgcaga agcggcatca gcaaaggcca ctgaagcgga aaaaagtgcc gcagccgcag
20221 agtcctcaaa aaacgcggcg gccaccagtg ccggtgcggc gaaaacgtca gaaacgaatg
20281 ctgcagcgtc acaacaatca gccgccacgt ctgcctccac cgcggccacg aaagcgtcag
20341 aggccgccac ttcagcacga gatgcggtgg cctcaaaaga ggcagcaaaa tcatcagaaa
20401 cgaacgcatc atcaagtgcc ggtcgtgcag cttcctcggc aacggcggca gaaaattctg
20461 ccagggcggc aaaaacgtcc gagacgaatg ccaggtcatc tgaaacagca gcggaacgga
20521 gcgcctctgc cgcggcagac gcaaaaacag cggcggcggg gagtgcgtca acggcatcca
20581 cgaaggcgac agaggctgcg ggaagtgcgg tatcagcatc gcagagcaaa agtgcggcag
20641 aagcggcggc aatacgtgca aaaaattcgg caaaacgtgc agaagatata gcttcagctg
20701 tcgcgcttga ggatgcggac acaacgagaa aggggatagt gcagctcagc agtgcaacca
20761 acagcacgtc tgaaacgctt gctgcaacgc caaaggcggt taaggtggta atggatgaaa
20821 cgaacagaaa agcccactgg acagtccggc actgaccgga acgccaacag caccaaccgc
20881 gctcagggga acaaacaata cccagattgc gaacaccgct tttgtactgg ccgcgattgc
20941 agatgttatc gacgcgtcac ctgacgcact gaatacgctg aatgaactgg ccgcagcgct
21001 cgggaatgat ccagattttg ctaccaccat gactaacgcg cttgcgggta aacaaccgaa
21061 gaatgcgaca ctgacggcgc tggcagggct ttccacggcg aaaaataaat taccgtattt
21121 tgcggaaaat gatgccgcca gcctgactga actgactcag gttggcaggg atattctggc
21181 aaaaaattcc gttgcagatg ttcttgaata ccttggggcc ggtgagaatt cggcctttcc
21241 ggcaggtgcg ccgatcccgt ggccatcaga tatcgttccg tctggctacg tcctgatgca
21301 ggggcaggcg tttgacaaat cagcctaccc aaaacttgct gtcgcgtatc catcgggtgt
21361 gcttcctgat atgcgaggct ggacaatcaa ggggaaaccc gccagcggtc gtgctgtatt
21421 gtctcaggaa caggatggaa ttaagtcgca cacccacagt gccagtgcat ccggtacgga
21481 tttggggacg aaaaccacat cgtcgtttga ttacgggacg aaaacaacag gcagtttcga
21541 ttacggcacc aaatcgacga ataacacggg ggctcatgct cacagtctga gcggttcaac
21601 aggggccgcg ggtgctcatg cccacacaag tggtttaagg atgaacagtt ctggctggag
21661 tcagtatgga acagcaacca ttacaggaag tttatccaca gttaaaggaa ccagcacaca
21721 gggtattgct tatttatcga aaacggacag tcagggcagc cacagtcact cattgtccgg
21781 tacagccgtg agtgccggtg cacatgcgca tacagttggt attggtgcgc accagcatcc
21841 ggttgttatc ggtgctcatg cccattcttt cagtattggt tcacacggac acaccatcac
21901 cgttaacgct gcgggtaacg cggaaaacac cgtcaaaaac attgcattta actatattgt
21961 gaggcttgca taatggcatt cagaatgagt gaacaaccac ggaccataaa aatttataat
22021 ctgctggccg gaactaatga atttattggt gaaggtgacg catatattcc gcctcatacc
22081 ggtctgcctg caaacagtac cgatattgca ccgccagata ttccggctgg ctttgtggct
22141 gttttcaaca gtgatgaggc atcgtggcat ctcgttgaag accatcgggg taaaaccgtc
22201 tatgacgtgg cttccggcga cgcgttattt atttctgaac tcggtccgtt accggaaaat
22261 tttacctggt tatcgccggg aggggaatat cagaagtgga acggcacagc ctgggtgaag
22321 gatacggaag cagaaaaact gttccggatc cgggaggcgg aagaaacaaa aaaaagcctg
22381 atgcaggtag ccagtgagca tattgcgccg cttcaggatg ctgcagatct ggaaattgca
22441 acgaaggaag aaacctcgtt gctggaagcc tggaagaagt atcgggtgtt gctgaaccgt
22501 gttgatacat caactgcacc tgatattgag tggcctgctg tccctgttat ggagtaatcg
22561 ttttgtgata tgccgcagaa acgttgtatg aaataacgtt ctgcggttag ttagtatatt
22621 gtaaagctga gtattggttt atttggcgat tattatcttc aggagaataa tggaagttct
22681 atgactcaat tgttcatagt gtttacatca ccgccaattg cttttaagac tgaacgcatg
22741 aaatatggtt tttcgtcatg ttttgagtct gctgttgata tttctaaagt cggttttttt
22801 tcttcgtttt ctctaactat tttccatgaa atacattttt gattattatt tgaatcaatt
22861 ccaattacct gaagtctttc atctataatt ggcattgtat gtattggttt attggagtag
22921 atgcttgctt ttctgagcca tagctctgat atccaaatga agccataggc atttgttatt
22981 ttggctctgt cagctgcata acgccaaaaa atatatttat ctgcttgatc ttcaaatgtt

23041 gtattgatta aatcaattgg atggaattgt ttatcataaa aaattaatgt ttgaatgtga
23101 taaccgtcct ttaaaaaagt cgtttctgca agcttggctg tatagtcaac taactcttct
23161 gtcgaagtga tatttttagg cttatctacc agttttagac gctctttaat atcttcagga
23221 attattttat tgtcatattg tatcatgcta aatgacaatt tgcttatgga gtaatctttt
23281 aattttaaat aagttattct cctggcttca tcaaataaag agtcgaatga tgttggcgaa
23341 atcacatcgt cacccattgg attgtttatt tgtatgccaa gagagttaca gcagttatac
23401 attctgccat agattatagc taaggcatgt aataattcgt aatcttttag cgtattagcg
23461 acccatcgtc tttctgattt aataatagat gattcagtta aatatgaagg taatttcttt
23521 tgtgcaagtc tgactaactt ttttatacca atgtttaaca tactttcatt tgtaataaac
23581 tcaatgtcat tttcttcaat gtaagatgaa ataagagtag cctttgcctc gctatacatt
23641 tctaaatcgc cttgtttttc tatcgtattg cgagaatttt tagcccaagc cattaatgga
23701 tcatttttcc atttttcaat aacattattg ttataccaaa tgtcatatcc tataatctgg
23761 tttttgtttt tttgaataat aaatgttact gttcttgcgg tttggaggaa ttgattcaaa
23821 ttcaagcgaa ataattcagg gtcaaaatat gtatcaatgc agcatttgag caagtgcgat
23881 aaatctttaa gtcttctttc ccatggtttt ttagtcataa aactctccat tttgataggt
23941 tgcatgctag atgctgatat attttagagg tgataaaatt aactgcttaa ctgtcaatgt
24001 aatacaagtt gtttgatctt tgcaatgatt cttatcagaa accatatagt aaattagtta
24061 cacaggaaat ttttaatatt attattatca ttcattatgt attaaaatta gagttgtggc
24121 ttggctctgc taacacgttg ctcataggag atatggtaga gccgcagaca cgtcgtatgc
24181 aggaacgtgc tgcggctggc tggtgaactt ccgatagtgc gggtgttgaa tgatttccag
24241 ttgctaccga ttttacatat tttttgcatg agagaatttg taccacctcc caccgaccat
24301 ctatgactgt acgccactgt ccctaggact gctatgtgcc ggagcggaca ttacaaacgt
24361 ccttctcggt gcatgccact gttgccaatg acctgcctag gaattggtta gcaagttact
24421 accggatttt gtaaaaacag ccctcctcat ataaaaagta ttcgttcact tccgataagc
24481 gtcgtaattt tctatctttc atcatattct agatccctct gaaaaaatct tccgagtttg
24541 ctaggcactg atacataact cttttccaat aattggggaa gtcattcaaa tctataatag
24601 gtttcagatt tgcttcaata aattctgact gtagctgctg aaacgttgcg gttgaactat
24661 atttccttat aacttttacg aaagagtttc tttgagtaat cacttcactc aagtgcttcc
24721 ctgcctccaa acgatacctg ttagcaatat ttaatagctt gaaatgatga agagctctgt
24781 gtttgtcttc ctgcctccag ttcgccgggc attcaacata aaaactgata gcacccggag
24841 ttccggaaac gaaatttgca tatacccatt gctcacgaaa aaaaatgtcc ttgtcgatat
24901 agggatgaat cgcttggtgt acctcatcta ctgcgaaaac ttgacctttc tctcccatat
24961 tgcagtcgcg gcacgatgga actaaattaa taggcatcac cgaaaattca ggataatgtg
25021 caataggaag aaaatgatct atattttttg tctgtcctat atcaccacaa aatggacatt
25081 tttcacctga tgaaacaagc atgtcatcgt aatatgttct agcgggtttg tttttatctc
25141 ggagattatt ttcataaagc ttttctaatt taacctttgt caggttacca actactaagg
25201 ttgtaggctc aagagggtgt gtcctgtcgt aggtaaataa ctgacctgtc gagcttaata
25261 ttctatattg ttgttctttc tgcaaaaaag tggggaagtg agtaatgaaa ttatttctaa
25321 catttatctg catcatacct tccgagcatt tattaagcat ttcgctataa gttctcgctg
25381 gaagaggtag ttttttcatt gtactttacc ttcatctctg ttcattatca tcgcttttaa
25441 aacggttcga ccttctaatc ctatctgacc attataattt tttagaatgg tttcataaga
25501 aagctctgaa tcaacggact gcgataataa gtggtggtat ccagaatttg tcacttcaag
25561 taaaaacacc tcacgagtta aaacacctaa gttctcaccg aatgtctcaa tatccggacg
25621 gataatattt attgcttctc ttgaccgtag gactttccac atgcaggatt ttggaacctc
25681 ttgcagtact actggggaat gagttgcaat tattgctaca ccattgcgtg catcgagtaa
25741 gtcgcttaat gttcgtaaaa aagcagagag caaaggtgga tgcagatgaa cctctggttc
25801 atcgaataaa actaatgact tttcgccaac gacatctact aatcttgtga tagtaaataa
25861 aacaattgca tgtccagagc tcattcgaag cagatatttc tggatattgt cataaaacaa
25921 tttagtgaat ttatcatcgt ccacttgaat ctgtggttca ttacgtctta actcttcata
25981 tttagaaatg aggctgatga gttccatatt tgaaaagttt tcatcactac ttagtttttt
26041 gatagcttca agccagagtt gtctttttct atctactctc atacaaccaa taaatgctga
26101 aatgaattct aagcggagat cgcctagtga ttttaaacta ttgctggcag cattcttgag
26161 tccaatataa aagtattgtg taccttttgc tgggtcaggt tgttctttag gaggagtaaa
26221 aggatcaaat gcactaaacg aaactgaaac aagcgatcga aaatatccct ttgggattct
26281 tgactcgata agtctattat tttcagagaa aaaatattca ttgttttctg ggttggtgat
26341 tgcaccaatc attccattca aaattgttgt tttaccacac ccattccgcc cgataaaagc
26401 atgaatgttc gtgctgggca tagaattaac cgtcacctca aaaggtatag ttaaatcact
26461 gaatccggga gcactttttc tattaaatga aaagtggaaa tctgacaatt ctggcaaacc
26521 atttaacaca cgtgcgaact gtccatgaat ttctgaaaga gttacccctc taagtaatga
26581 ggtgttaagg acgctttc pos26598
